# Nucleus Accumbens Proteome Disbalance in an Adolescent Mouse Model of Schizophrenia and Nicotine Misuse Comorbidity

**DOI:** 10.3390/biomedicines13040901

**Published:** 2025-04-08

**Authors:** Thainá Pereira Souza, Andrés Rodríguez-Vega, Ana Carolina Dutra-Tavares, Keila A. Semeão, Claudio Carneiro Filgueiras, Anderson Ribeiro-Carvalho, Alex Christian Manhães, Yael Abreu-Villaça

**Affiliations:** 1 Laboratório de Neurofisiologia, Departamento de Ciências Fisiológicas, Instituto de Biologia Roberto Alcantara Gomes, Universidade do Estado do Rio de Janeiro (UERJ), Av. Prof. Manuel de Abreu 444, 5 Andar—Vila Isabel, Rio de Janeiro 20550-170, RJ, Brazil; thainasouza2@gmail.com (T.P.S.); andresve22@gmail.com (A.R.-V.); brainherkeila@gmail.com (K.A.S.); ccfilg@yahoo.com.br (C.C.F.); ac_manhaes@yahoo.com.br (A.C.M.); 2 Departamento de Ciências Biomédicas e Saúde, Instituto de Biologia Roberto Alcantara Gomes, Universidade do Estado do Rio de Janeiro (UERJ), Cabo Frio 28905-320, RJ, Brazil; dutratavaresana@gmail.com; 3 Departamento de Ciências, Faculdade de Formação de Professores, Universidade do Estado do Rio de Janeiro (UERJ), São Gonçalo 24435-005, RJ, Brazil; ribeiro_carvalho@yahoo.com.br

**Keywords:** mental disorder, nicotine addiction, proteome, E-cigarette, mass spectrometry, prodrome, co-exposure, mesolimbic system

## Abstract

**Background/Objectives**: Schizophrenia and nicotine misuse are a comorbid condition that frequently develops during adolescence. Considering the role of the nucleus accumbens (NAcc) as a common neurobiological substrate for these psychiatric disorders, label-free proteomics was employed to identify NAcc deregulated proteins in male and female mouse models of schizophrenia with a history of adolescent nicotine exposure. **Methods**: Phencyclidine was used to model schizophrenia, and minipump infusions were used to model nicotine misuse. **Results**: Enrichment Reactome pathway and protein–protein interaction analyses showed that the cytoskeleton and associated synaptic plasticity mechanisms, energy metabolism, and nervous system development were affected in both sexes. In particular, Ncam1 (Neural cell adhesion molecule 1) could be of interest as a candidate marker of synaptic plasticity disbalance. Its deregulation in the NAcc of both sexes suggests that it lies at the core of the comorbidity pathophysiology. When considering sex-selective effects, Cs (Citrate synthase) and Mapk3 (Mitogen-activated protein kinase 3) were identified as exclusively deregulated in female and male mice, respectively. Since both proteins were previously shown to be exclusively deregulated in the medial prefrontal cortex of co-modeled mice, a common mesocortical and mesolimbic system effect can be inferred, supporting the role of aberrant energy metabolism and synaptic plasticity in the comorbidity model. **Conclusions**: The current data provide insights into the NAcc proteome disbalance in an adolescent preclinical model of combined schizophrenia and nicotine misuse, pointing to relevant pathways and early markers of the comorbidity.

## 1. Introduction

The nucleus accumbens (NAcc) is a major projection target of the mesolimbic dopaminergic system. This component of the striatum is mainly composed of GABAergic medium spiny neurons [1] and receives dopaminergic inputs from the ventral tegmental area (VTA) and glutamatergic projections from the prefrontal cortex, hippocampus, and amygdala, among others. Some of the most relevant outputs, in turn, reach the ventral pallidum, the VTA, the hypothalamus, and the substantia nigra [2,3,4]. These interconnected regions collectively contribute to the reinforcement of rewarding behaviors, the regulation of motivation, and decision making [5,6,7,8,9]. Adolescence is a critical period for the maturation of the NAcc. During adolescence, synaptic pruning is increased in mesolimbic regions, contributing to neural circuit refinement and communication efficiency [10,11,12]. In the NAcc, the activity of dopaminergic synapses peaks during adolescence when compared to both earlier postnatal periods, making this region particularly sensitive to stimuli [10,11].

These and other neurobiological maturation events might underlie the increased susceptibility in adolescence to drugs of abuse [13,14] and may also be relevant to other mental health disorders [15,16]. Among psychiatric disorders, schizophrenia (SCHZ) stands out as a severe chronic condition. It shows sexual dimorphic differences in several aspects, including prevalence, clinical presentation, and treatment efficacy [17], and is characterized by disruptions in thought processes, perceptions, emotional responsiveness, and social interactions [18,19], which profoundly impact an individual’s ability to function in daily life. Mesolimbic aberrant dopaminergic signaling is classically associated with SCHZ-positive symptoms, such as hallucinations and delusions [20]. Recent evidence further shows that positive, negative, and cognitive SCHZ symptoms are interrelated, pointing to an additional involvement of the mesolimbic system in negative and cognitive outcomes [21,22,23]. Regarding substance use disorders, nicotine, as the main neuroactive component of tobacco products, is both highly addictive and harmful. As a result, tobacco smoking continues as the leading cause of preventable death worldwide [24]. The mesolimbic system is a central mediator of nicotine addictive effects. This drug of abuse’s actions in the VTA increase dopamine release in the NAcc [25,26]. This dopamine surge is more robust during adolescence, contributing to heightened reinforcement responses and associated neuroplasticity events, ultimately increasing the risk of nicotine misuse [25,26].

SCHZ is associated with a high risk of comorbid conditions, including substance use disorders. In particular, nicotine misuse and abuse are notably frequent in SCHZ patients, with smoking rates in this population far exceeding those in the general population [27,28]. The association between SCHZ and nicotine misuse develops during adolescence. Milder positive, cognitive, and negative symptoms of SCHZ frequently emerge during this period, before a formal diagnosis is made [29,30,31]. Adolescence also coincides with experimentation and progression to misuse of both traditional combustible tobacco products [32] and electronic nicotine delivery systems (ENDSs) [33,34]. Supporting these data, the prevalence of tobacco consumption in the prodromal phase of SCHZ ranges from 17% to 46% [35] and stands between 59% and 78% among patients experiencing the first episode of psychosis, when compared to healthy controls [36,37].

The underlying reasons for this comorbidity are still under investigation. Tobacco smoking has been linked to an increased risk of SCHZ and more severe symptoms. It is also known that it contributes to a reduced life expectancy and overall health deterioration in SCHZ patients [38,39]. Despite that, paradoxically, beneficial effects of smoking have also been observed, and nicotinic agents might offer a potential strategy for alleviating SCHZ symptoms [38,39]. These conflicting data indicate the need for a better understanding of the mechanisms that lead to the comorbidity. In this regard, the investigation of SCHZ and nicotine misuse outcomes in the mesolimbic system might provide valuable information.

Even though adolescence is a crucial time window for both nicotine misuse and SCHZ, ethical concerns and technical and analytical limitations hamper the access to human data during this period of comorbid susceptibility [40,41]. In this context, the use of animal models stands out as an alternative of translational significance. Our research group has used phencyclidine in mice to model SCHZ during adolescence and young adulthood [42,43,44,45]. This non-competitive NMDA receptor antagonist is a well-known psychomimetic drug widely used in experimental studies [46,47] that evokes SCHZ-like behaviors [48], causing deficits in prepulse inhibition [49] and neurochemical dysfunctions similar to those identified in SCHZ [50]. Regarding nicotine exposure, osmotic minipumps are a well-established, low stress model that provides a consistent dose and a controlled duration of administration [51,52]. It has been shown to interfere with SCHZ-like behavioral outcomes [42,43] and to alter the frontal cortex proteomic profile [45] during adolescence in mice.

Techniques such as quantitative proteomics have provided relevant information on the underlying molecular alterations of SCHZ [53,54] and nicotine misuse [55]. However, efforts directed to the identification of pathways and biomarkers involved in these disorders’ comorbidity are scant, particularly during the period of its establishment. Here, by using the aforementioned animal models, we preclinically identified factors associated with the early course of the SCHZ + nicotine misuse comorbid condition. The NAcc was chosen due to evidence of its role as a common neurobiological substrate for the interplay between SCHZ and nicotine misuse [3]; males and females were analyzed separately due to previous evidence of relevant sex differences in SCHZ [17] and in the comorbidity [45]. Subchronic phencyclidine administration in adolescent mice was combined with a short period of nicotine exposure [42]. We hypothesized that the analysis of the NAcc would reveal relevant pathways and early key markers of the combined insult that may help explain the association between SCHZ and nicotine misuse.

## 2. Materials and Methods

### 2.1. Materials

Ketamine (CEVA, Paulínea, SP, Brazil), xylazine (Syntec, Barueri, SP, Brazil), flunixin (UCB/VET, Jaboticabal, SP, Brazil), and enrofloxacin (VENCO, Londrina, PR, Brazil) were specific for veterinary use. Nicotine-free base, dithiothreitol (DTT), iodoacetamide (IAA), formic acid, sodium deoxycholate, and triethylammonium bicarbonate buffer (TEAB) were purchased from Sigma Chemical Co. (St. Louis, MO, USA). Phencyclidine was purchased from Alomone Labs (Jerusalem, Israel). Osmotic minipumps came from Alzet (Cupertino, CA, USA). Promega Biotecnologia do Brasil (São Paulo, SP, Brazil) was the source of trypsin, and Thermo Fisher Scientific (Waltham, MA, USA) was the source of the POROS 20 R2 resin column and the Qubit protein assay kit. The nanoEase Symmetry C18 (5 µm, 180 µm × 20 mm) TRAP column, nanoAcquity BEH130 C18 (1.7 µm, 75 µm × 100 mm) reversed-phase column, [Glu1]-Fibrinopeptide B human (GFP), and Total Recovery vials were purchased from Waters Corps (Milford, MA, USA). Acetonitrile came from Merck HGaA (Darmstadt, Germany).

### 2.2. Animals and Treatment

The experimental procedures were approved by the Institute of Biology/UERJ Ethical Committee for Animal Research (protocol No: CEUA/033/2018), minimizing the number of animals used and avoiding animal suffering, in accordance with Brazilian Law No. 11.794/2008. All mice were kept in our animal facility housed in groups of 2–5 at 21–22 °C on a 12 h light/dark cycle (lights on at 1:00 a.m.). Access to food and filtered water was ad libitum. Animals were derived from a C57BL/6 colony maintained at the Universidade Federal Fluminense (Niteroi, Brazil) for over 60 generations.

Mice were exposed to a nicotine solution or milli-Q water from postnatal day (PN) 37 to PN44. On PN36, osmotic minipumps (model 1007d) were filled either with milli-Q water or nicotine-free base diluted in milli-Q water (pH 6.0) so as to deliver an initial dose rate of 24 mg/Kg of nicotine per day. At PN37, mice were anesthetized with xylazine (20 mg/Kg, i.p.) and ketamine (100 mg/Kg, i.p.), a small area on the back was shaved, an incision was made to allow s.c. insertion of the minipump, and the incisions were sutured. For pain management and infection prevention, the mice were given s.c. injections of Flunixin (2.5 mg/Kg) and Enrofloxacin (2.5 mg/Kg) right after the end of surgery and on the following day. Animals were allowed to recover from surgery in their home cages. The period of exposure to nicotine was intended to parallel human exposure during adolescence, a key period for the initiation of tobacco and ENDS consumption [33,56,57]. The dose rate used in the current study results in nicotine serum levels [51] that are within the range of those found in smokers [58,59].

From PN38 to PN52, the animals were exposed daily to phencyclidine, an NMDA receptor antagonist used to model SCHZ, at a dose of 2.5 mg/Kg (s.c.) [60,61]. On the last day of phencyclidine exposure (PN53), the dose used was 10 mg/Kg (s.c.) [62]. The period of phencyclidine exposure was chosen based on evidence that the prodromal stage of the disorder begins early, still during adolescence [29,30,31], and that the first psychotic event in SCHZ frequently occurs between late adolescence and early adulthood [30,63]. Control mice received a saline solution (NaCl 0.9%, s.c.). Accordingly, there were four experimental groups: CT (control group), PCP (phencyclidine-exposed group), NIC (nicotine-exposed group), and PCPNIC (phencyclidine- and nicotine-exposed group).

At PN53, after the end of nicotine exposure but still during the period of exposure to phencyclidine, the mice were decapitated, the brains were flash-frozen in liquid nitrogen, and stored at −76 °C. The 9-day interval between the end of nicotine exposure and euthanasia aimed to assess the lasting effects of nicotine without the presence of confounding factors associated with acute withdrawal signs [64,65,66]. Samples were punched from the frozen brains using a cryostat (Hyrax C25, Zeiss, Oberkochen, Germany) and a cylindric brain puncher (internal diameter 1.0 mm). The length and location of the punches followed the coordinates described in the Paxinos and Franklin stereotaxic atlas [67]: 0.48 mm of length (starting at 1.34 mm anterior to Bregma), aiming for the NAcc. The samples were frozen at −76 °C for posterior proteomics analysis.

### 2.3. Protein Extraction

In order to guarantee minimum sample concentration for the analysis, pools of samples from different mice were used instead of individual samples. Punches of the NAcc from two animals within each experimental condition and sex produced one sample pool. Four sample pools of each experimental group and sex were used, resulting in four biological replicates and three technical replicates. Each sample pool was extracted with 500 μL of buffer (50 mM TEAB, pH 8.5, 0.1% sodium deoxycholate). The samples were briefly vortexed and then incubated in an ultrasonic bath (SolidSteel, Piracicaba, SP, Brazil) for 30 min at 6 °C. After centrifugation at 18,400× *g* for 20 min at 4 °C (Eppendorf, centrifuge 5427R, Hamburg, Germany), the supernatants were transferred to new identified tubes. The extraction was performed twice, and the respective supernatants were merged. Acetonitrile with 0.1% formic acid was added to the supernatant for protein precipitation in a 4:1 proportion (*v*/*v*), and the samples were centrifuged at 18,400× *g* for 10 min and 4 °C and washed twice with cold acetone. Proteins were resuspended in TEAB 50 mM, pH 8.5, dosed using a Qubit^®^ fluorometer (Invitrogen, Waltham, MA, USA), and processed as described previously [45,68].

### 2.4. Protein Digestion and nanoULPC-MSE Analysis

Forty micrograms of proteins was treated with 0.01% sodium deoxycholate, reduced by the addition of 10 mM DTT at 56 °C for 30 min, and alkylated with IAA 20 mM at 25 °C for 30 min. Afterward, the samples were digested with sequencing grade trypsin in a 1:100 trypsin/protein ratio in TEAB 50 mM, pH 8.5, for 18 h at 37 °C under agitation on a Thermomixer Comfort (Thermo Fischer Scientific, Waltham, MA, USA). Peptides were desalinated and concentrated through reversed-phase chromatography with a POROS 20 R2 resin column and eluted with 7:3 acetonitrile/water with 0.05% formic acid. The peptides were dried, resuspended in 0.1% formic acid, quantified (Qubit^®^ protein assay kit), and the supernatants were transferred to Waters Total Recovery vials.

The nanoUPLC analysis of tryptic peptides was carried out using a nanoACQUITY UPLC system (Waters Corp., Milford, MA, USA) coupled to a Synapt G2-Si high-definition mass spectrometer (Waters Corp., Manchester, UK). The chromatographic system was equipped with a nanoEase Symmetry C18 (5 µm, 180 µm × 20 mm) TRAP column and a nanoAcquity BEH130 C18 (1.7 µm, 75 µm × 100 mm) reversed-phase column. Mobile phase A consisted of water, and mobile phase B consisted of acetonitrile, both with 0.1% (*v*/*v*) formic acid. The peptides were separated by Mass Spectrometry Experimentation (MSE) chromatographic methods (gradient of 3 to 50% B for 60 min; followed by a cleaning column gradient of 50 to 85% B for 1 min; maintained in 85% B for 5 min; then from 85 to 3% B for 1 min). The flow rate was 450 ηL·min^−1^. The analytical column temperature was maintained at 35 °C, and the sample manager temperature was 9 °C. The lock mass was derived from the auxiliary pump using a constant flow rate of 500 ηL·min^−1^ at a concentration of 200 fmol of GFP to the reference sprayer of the NanoLockSpray source.

The mass spectrometer was operated in resolution mode for all measurements. All analyses were carried out using nano-electrospray ionization in positive ion mode (nanoESI+) and a NanoLockSpray (Waters, Manchester, UK) ionization source. The lock mass channel was sampled at a frequency of 30 s. The ion source block temperature was set to 100 °C, and the capillary voltage was set to 3 kV. The time-of-flight analyzer of the mass spectrometer was calibrated with a MS/MS spectrum of GFP solution. The final instrument calibration was obtained using the signal of GFP double charged precursor ion [M + 2H]^2+^ = 785.8426. The exact mass retention time from multiplexed data-independent acquisition (DIA) scanning MS analyses (MSE) was collected in an alternating low-energy and elevated-energy acquisition mode. In the elevated-collision-energy mode, the collision energy was increased from 15 to 55 eV applied to the trap “T-wave” collision-induced dissociation cell with argon gas. The radiofrequency was adjusted such that the ions were effectively acquired from *m*/*z* 50 to 2000, which ensured that any masses less than 50 *m*/*z* observed in the elevated-energy spectra were only derived from dissociations in the collision cell.

### 2.5. Data Processing

In order to identify and quantify peptides and proteins, the MS data were processed and searched using the Progenesis QI for Proteomics version 4.2 (Nonlinear Dynamics, Milford, MA, USA) (for a detailed description of the methods used to establish the normality and significance of data, refer to Supplementary Material File S1). The UNIPROT protein databank release 2024 (http://www.uniprot.org, accessed on 17 September 2024) with specific annotations for *Mus musculus* was utilized. A reverse database was generated for monitoring the false positive rate (false discovery rate—FDR). The parameters for database searching were tryptic peptides with a maximum of two missed cleavages, maximum protein mass of 650 kDa, carbamidomethylation of cysteine as a fixed modification, and oxidation of methionine as variable modifications. The parameters set as default were a peptide mass error tolerance of 10 ppm, fragment mass error tolerance of 20 ppm, and maximum of 1% of the FDR. At the protein level, the criteria included the detection of at least two fragment ions per peptide, three or more fragments per protein, and the determination of at least two peptides per protein. For protein identification, both errors smaller than 20 ppm and scores higher than 10 were applied. Relative quantification was determined from the absolute intensities obtained and considered the Hi3 (Top3)-based quantitation method [69]. Samples were compared in pairs to obtain expression data (CT, PCP, NIC, and PCPNIC groups), and the following filters were used: coefficient of variance (maximum CV of 30%), max fold change (>1.5), and analysis of variance (*p* < 0.05). Only coexisting proteins in both conditions and proteins that were present in at least 3 replicates (3/4) were considered to compare the conditions.

Reactome pathways analysis was performed using the online Protein Analysis Through Evolutionary Relationships version 19.0 (PANTHER) classification system (http://www.pantherdb.org, accessed on 1 October 2024) [70]. The jvenn online tool (http://jvenn.toulouse.inra.fr/app/index.html, accessed on 15 October 2024) was used to draw Venn diagrams to visualize all possible intersections among proteome datasets. Protein–protein interaction analysis was performed using STRING version 12.0 (https://string-db.org, accessed on 4 November 2024) with the minimum confidence level accepted for interactions set to medium (0.4).

PCP, NIC, and PCPNIC were compared to CT mice aiming to identify the deregulated proteins for each experimental group. This approach allowed the identification of specific contributions of phencyclidine exposure and nicotine history to the profile of PCPNIC deregulated proteins, represented as proteins commonly deregulated in the PCP and PCPNIC groups, and in the NIC and PCPNIC ones, respectively. Proteins deregulated in all three drug-exposed groups indicate common contributions of phencyclidine and nicotine to the PCPNIC deregulated profile. The exclusive proteins, defined as the ones deregulated only in a given experimental group, were also identified and provide relevant information: Proteins exclusively deregulated in the PCPNIC group correspond to the ones differentially expressed as a result of the interactive events of nicotine and phencyclidine. Regarding exclusively deregulated proteins identified in the NIC and PCP groups, those point to alterations caused either by nicotine history or phencyclidine that are not relevant to the outcomes of the combined insult; all related information is provided in the Supplementary Material (Supplementary Material File S2—Sheets S12–S15). To characterize the relevance of the changes in the NAcc proteome, in addition to submitting the data to Reactome pathways analysis, both commonly and exclusively deregulated proteins were plotted in interactome maps.

The mass spectrometry proteomics data have been deposited in the Proteo-meXchange Consortium via the PRoteomics Identification Database (PRIDE) partner repository (http://www.ebi.ac.uk/pride, accessed on 12 February 2025) with the dataset identifier PXD060733 and 10.6019/PXD060733.

## 3. Results

A total of 3958 unique peptides that corresponded to 338 proteins were identified using a minimum of 2 peptides per protein and the presence in at least 2 of the replicate analyses. Supplementary Material File S2—Sheets S1 and S2 show a list of peptides containing identifiers for all NAcc proteins. These proteins were also ranked based on their abundance relative to their total independent spectral counts.

Studies in SCHZ patients show sex differences in SCHZ prevalence, symptom domains, and severity, which, to some extent, are replicated in animal models [17,71]. Accordingly, here, for the analyses, mice were a priori separated by sex. Most proteins (85.4%) were differentially expressed in only one sex (Supplementary Material File S2—Sheet S3). Additionally, for both males and females, changes in protein abundance depended on the drugs the mice were exposed to, which corroborates the importance of considering phencyclidine and nicotine as independent factors in the analysis.

### 3.1. Proteomic Profile of the NAcc of Mouse Models of SCHZ, Nicotine Misuse, and Their Co-Morbidity When Compared to Controls

**Figure 1:**
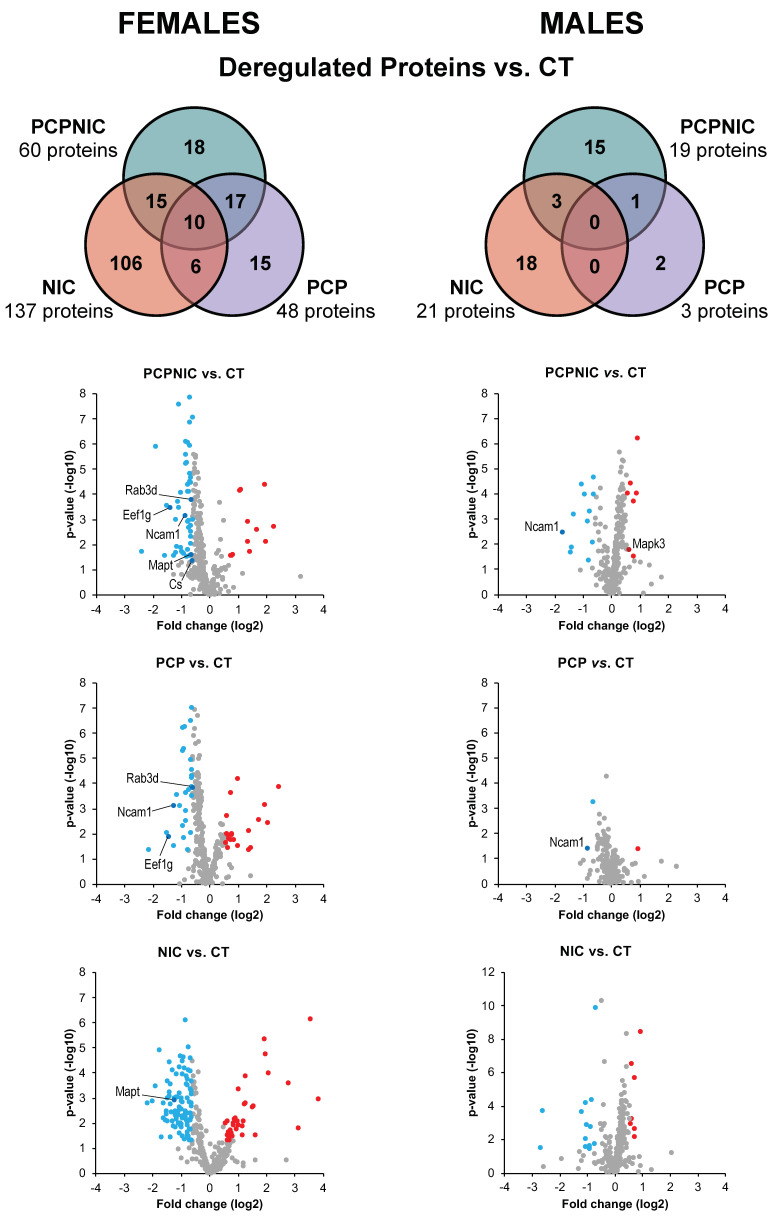
Venn diagrams (top panels) showing deregulated proteins in the NAcc of female (**left**) and male (**right**) mouse models of SCHZ (PCP), nicotine misuse (NIC), or the comorbidity (PCPNIC) when compared to controls (CT). Volcano plots (lower panels) provide a general picture of fold change magnitude (*x*-axis) and *p*-values (*y*-axis) of the dataset. Blue dots indicate downregulated proteins, red dots indicate upregulated ones, gray dots indicate proteins that did not reach deregulation criteria (>1.5 fold change and *p* < 0.05). Candidate markers of the comorbidity are identified and will be discussed below.

The results from comparisons between the CT and the other groups (PCP, NIC, and PCPNIC) are shown as Venn diagrams (Figure 1). Females showed a higher number of proteins that were differentially expressed when compared to males. In both sexes, most proteins were downregulated. In the PCPNIC group, 60 proteins were deregulated in females (48 downregulated, 12 upregulated), and 19 were deregulated in males (12 downregulated, 7 upregulated). PCP females presented 48 deregulated proteins (28 downregulated, 20 upregulated), while in males, 3 proteins were identified (2 downregulated, 1 upregulated). The NIC group showed the highest number of deregulated proteins in both females (137 proteins; 100 downregulated, 37 upregulated) and males (21 proteins; 14 downregulated, 7 upregulated). Supplementary Material File S2—Sheets S4–S9 provide detailed information regarding the aforementioned deregulated proteins.

### 3.2. Exclusively Deregulated Proteins

Exclusive proteins of the PCPNIC group are those differentially expressed only in this experimental group (Figure 1). The analysis of PCPNIC mice identified 18 exclusively deregulated proteins in females (16 downregulated, 2 upregulated) and 15 proteins in males (9 downregulated, 6 upregulated). Detailed information on the deregulated proteins involved in each comparison can be found in Supplementary Material File S2—Sheets S10 and S11.

**Figure 2:**
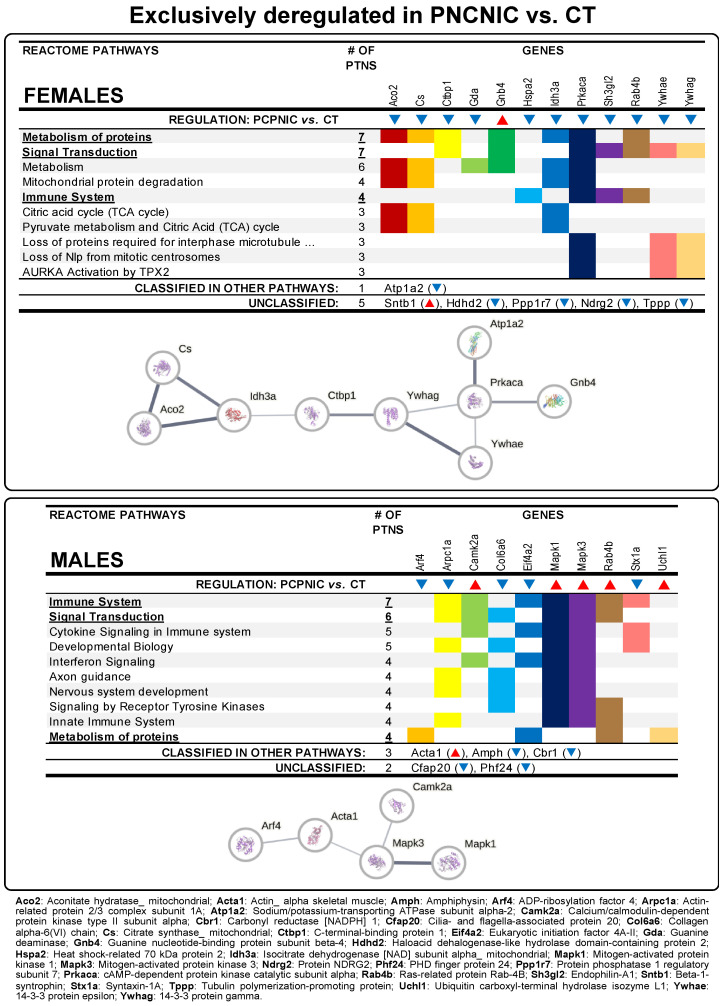
Data from exclusively deregulated proteins in the NAcc of female and male mouse models of the comorbidity (PCPNIC) when compared to controls (CT). The panels show top 10 Reactome pathways. Pathways in bold and underlined were shared between sexes. ▼ indicates that a given protein is downregulated in PCPNIC mice when compared to CT ones, while ▲ indicates that a given protein is upregulated. In the interaction networks, circles represent gene names, lines represent the interaction between proteins, and the results within the circle represent the structure of proteins. The proteins were analyzed by STRING, and the network was built using ‘medium confidence’ (0.4) interaction score. Each node represents a protein, and the line thickness indicates the strength of data support.

Among the 10 more frequent Reactome pathways identified in PCPNIC mice, 4 (signal transduction, signaling by receptor tyrosine kinases, immune system, and metabolism of proteins) were affected in both males and females (Figure 2). Mitochondrial protein degradation, pyruvate metabolism and tricarboxylic acid (TCA) cycle, and other pathways related to mitotic cell division were present among the other top Reactome pathways only in females (Figure 2 upper panel). As for males, other signaling-related pathways (cytokine signaling in immune system and interferon signaling) and pathways involved in the development of the nervous system (axon guidance and nervous system development) were enriched (Figure 2 lower panel). Supplementary Material File S3, Figure S1, provides the complete list of Reactome pathways for each sex.

To further understand the impact of nicotine and phencyclidine interactions in the profile of PCPNIC deregulated proteins in the NAcc of adolescent mice, those proteins were accommodated in interactome maps. The female interactome map consisted of nine deregulated proteins grouped into interconnected subnetworks (Figure 2 upper panel). In males, the interactome map was composed of five proteins (Figure 2 lower panel).

### 3.3. Commonly Deregulated Proteins

The Reactome pathways analyses of proteins commonly deregulated for different comparisons and their interactome maps are described below. Due to the smaller number of deregulated proteins in males, only commonly deregulated proteins in females formed interactome maps.

**Figure 3:**
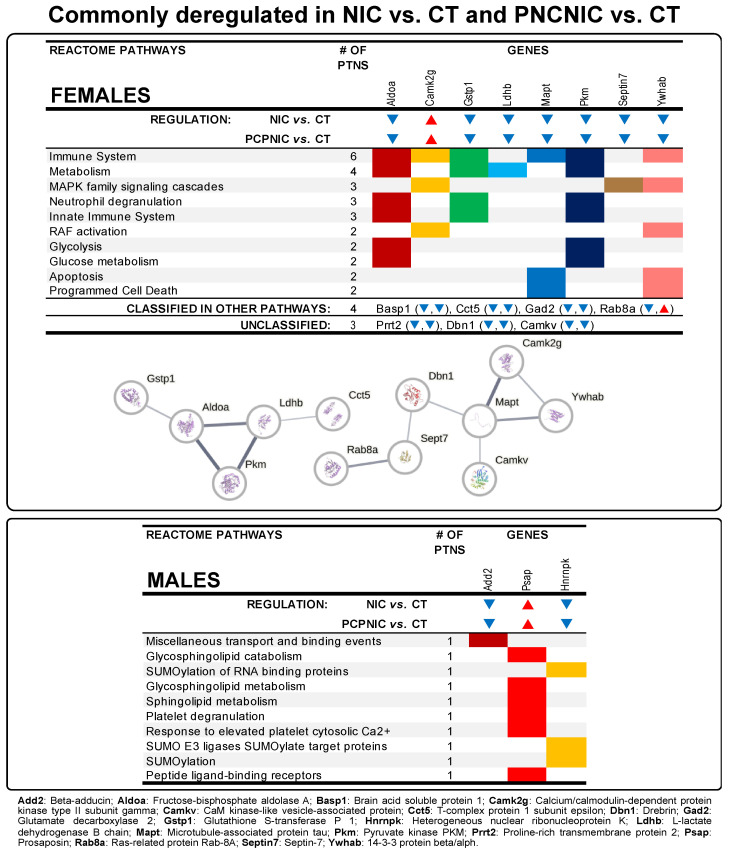
Data from proteins commonly deregulated in the NAcc of female and male mouse models of nicotine misuse (NIC) and the comorbidity (PCPNIC), when compared to controls (CT). The panels show top 10 Reactome pathways.▼indicates that a given protein is downregulated in NIC and PCPNIC mice when compared to CT ones, while ▲ indicates that a given protein is upregulated. In the interaction network, circles represent gene names, lines represent the interaction between proteins, and the results within the circle represent the structure of proteins. The proteins were analyzed by STRING, and the network was built using ‘medium confidence’ (0.4) interaction score. Each node represents a protein, and the line thickness indicates the strength of data support.

The specific contributions of nicotine history to the NAcc profile of PCPNIC deregulated proteins in females included 15 proteins that were commonly deregulated in NIC and PCPNIC when compared to CT mice (Figure 1; Supplementary Material File S2—Sheets S16 and S17). As for males, 3 commonly deregulated proteins were identified (Figure 1). The Reactome analyses indicated that none of the 10 more frequent pathways were shared between females and males (Figure 3). In females, those pathways were associated with the immune response (immune system and innate immune system), glucose metabolism (metabolism, glucose metabolism and glycolysis), mitogen-activated protein kinase (MAPK) signaling (MAPK family signaling cascades, RAF activation), and cell death (apoptosis, programmed cell death, neutrophil degranulation) (Figure 3 upper panel). The interactome map of commonly deregulated proteins in NIC and PCPNIC female mice included 12 of these proteins, which fit into two independent subnetworks (Figure 3 upper panel). In males, protein SUMOylation (SUMOylation of RNA binding protein, SUMO E3 ligases SUMOylate target proteins), lipid metabolism (glycosphingolipid catabolism and metabolism, sphingolipid metabolism), transport and binding (miscellaneous transport and binding events, peptide ligand-binding receptors), and others related to platelet responses (platelet degranulation, response to elevated platelet cytosolic Ca^++^) were the most relevant pathways (Figure 3 lower panel).

**Figure 4:**
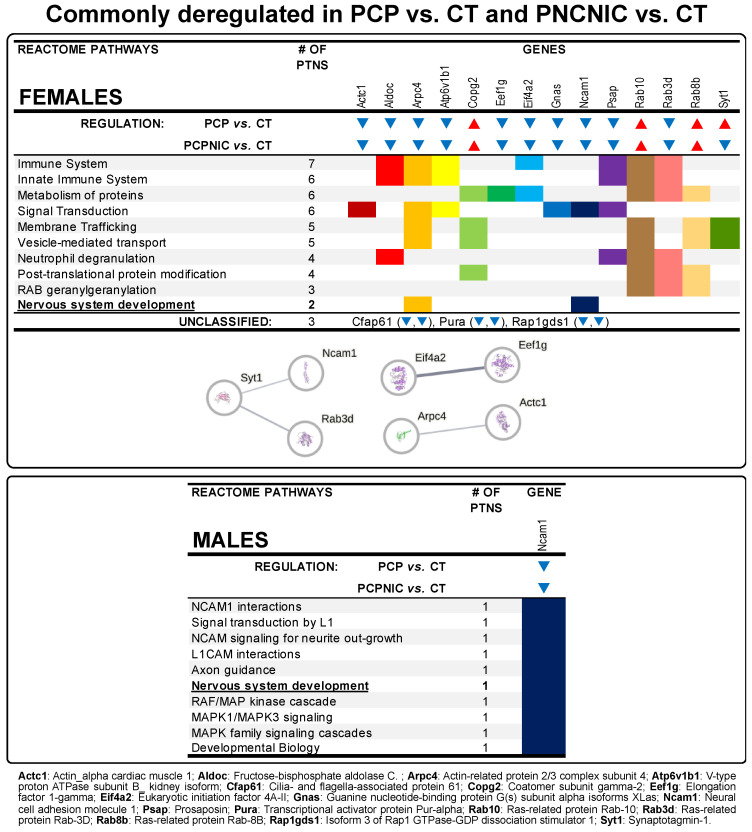
Data from proteins commonly deregulated in the NAcc of female and male mouse models of schizophrenia (PCP) and the comorbidity (PCPNIC), when compared to controls (CT). The panels show top 10 Reactome pathways.▼indicates that a given protein is downregulated in PCP and PCPNIC mice when compared to CT ones, while ▲ indicates that a given protein is upregulated. In the interaction network, circles represent gene names, lines represent the interaction between proteins, and the results within the circle represent the structure of proteins. The proteins were analyzed by STRING, and the network was built using ‘medium confidence’ (0.4) interaction score. Each node represents a protein, and the line thickness indicates the strength of data support.

The specific contributions of phencyclidine exposure to the NAcc profile of PCPNIC deregulated proteins are represented by proteins commonly deregulated in the PCP and PCPNIC groups (Figure 1; Supplementary Material File S2—Sheets S18 and S19). This analysis identified 17 proteins in females and 1 protein in males. The Reactome analysis indicated that among the top 10 enriched pathways, nervous system development was common to both males and females (Figure 4). In females, immune and innate immune systems, post-translational modification and metabolism of proteins, signal transduction, vesicle-mediated transport, neutrophil degranulation, and membrane trafficking-related pathways (membrane trafficking, RAB geranylgeranylation) stood out (Figure 4 upper panel). As for the interactome map of commonly deregulated proteins in PCP and PCPNIC males, 7 of these 17 deregulated proteins were grouped into three independent subnetworks (Figure 4 upper panel). In males, the sole protein identified was implicated in several related Reactome pathways relevant to the development of the nervous system, hemophilic and heterophilic protein–protein interactions, and cell signaling (NCAM and L1CAM interactions, signal transduction by L1, NCAM signaling for neurite outgrowth, axon guidance, nervous system development, RAF/MAP kinase cascade, MAPK1/MAPK3 signaling, MAPK family signaling cascades, and developmental biology) (Figure 4 lower panel).

**Figure 5:**
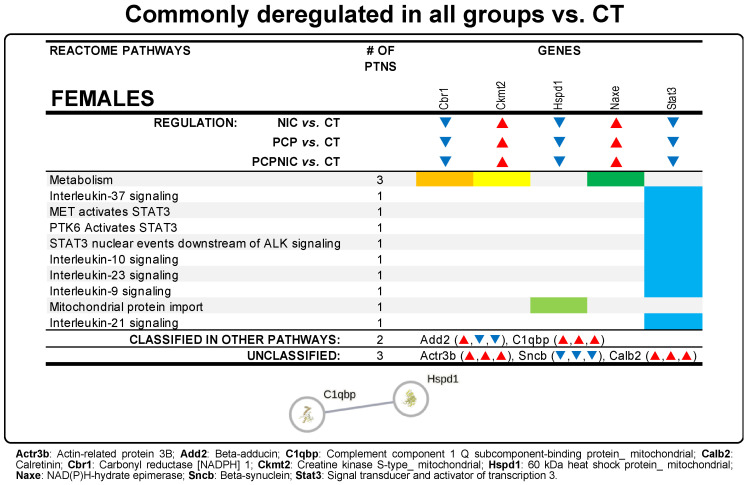
Data from proteins commonly deregulated in the NAcc of female mouse models of schizophrenia (PCP), nicotine misuse (NIC), and the comorbidity (PCPNIC), when compared to controls (CT). The panel shows top 10 Reactome pathways.▼indicates that a given protein is downregulated in NIC, PCP, and PCPNIC mice when compared to CT ones, while ▲ indicates that a given protein is upregulated. In the interaction network, circles represent gene names, lines represent the interaction between proteins, and the results within the circle represent the structure of proteins. The proteins were analyzed by STRING, and the network was built using ‘medium confidence’ (0.4) interaction score. Each node represents a protein, and the line thickness indicates the strength of data support.

Proteins deregulated in all three drug-exposed groups indicate common contributions of PCP and NIC to the PCPNIC deregulated profile (Figure 1). In females, 10 proteins were commonly deregulated in PCPNIC, NIC, and PCP when compared to CT mice (Figure 5; Supplementary Material File S2—Sheet S20). Among the enriched Reactome pathways, those involving interleukin signaling stood out [interleukin-9, interleukin-10, interleukin-21, interleukin-23, and interleukin-37 signaling; MET-activated signal transducers and activators of transcription 3 (STAT3); PTK6 activates STAT3; STAT3 nuclear events downstream of ALK signaling] (Figure 5). The map for commonly deregulated proteins in PCPNIC, NIC, and PCP when compared to CT mice was composed of a subnetwork that included 2 of the 10 deregulated proteins (Figure 5). There were no commonly deregulated proteins in males.

## 4. Discussion

The current study shows for the first time disrupted expression of proteins in the NAcc of female and male adolescent mouse models of SCHZ with and without a history of nicotine exposure. The comparison between differentially expressed proteins in PCP (PCP vs. CT) and PCPNIC (PCPNIC vs. CT) mice shows a higher number of deregulated proteins in the NAcc of co-exposed mice, suggesting a worsening effect of nicotine history on mouse models of SCHZ, mostly in males. In contrast, despite the short-term period of exposure, nicotine led to a more severe protein deregulation (NIC vs. CT) than that caused both by PCP and PCPNIC. The higher number of proteins differentially expressed in NIC mice when compared to PCPNIC suggests that in co-exposed mice, phencyclidine and nicotine interacted, leading to less severe effects than nicotine history only, an effect that was particularly evident in females. Sex differences were also identified during the analysis of commonly and exclusively deregulated proteins, which resulted in sex-specific Reactome pathways and protein–protein interaction networks. Altogether, these data align with previous findings of sex differences in SCHZ patients [72,73,74,75] and animal models of SCHZ [45,71,76], as well as in smokers [77] and rodents exposed to nicotine [64,78,79]. Of note, despite the sex-selective effects in the deregulated proteins, as elaborated below, targets such as the cytoskeleton and associated synaptic plasticity mechanisms, as well as energy metabolism and nervous system development, were clearly identified in both female and male models of the comorbid condition. This suggests that, to some extent, the core mechanisms of the comorbidity are similar in both sexes.

### 4.1. Exclusively Deregulated Proteins in PCPNIC Mice

Exclusively deregulated proteins in PCPNIC mice may indicate subthreshold effects of nicotine history and phencyclidine that, when combined, lead to the disrupted expression of proteins. However, it is also possible that interactive events between insults play a role, which corroborates previous data [43,44,45,80]. Reactome pathways of these proteins were enriched for metabolism of proteins, signal transduction, signaling by receptor tyrosine kinases, and immune system in both males and females. However, female-only pathways including pyruvate metabolism and TCA cycle and mitotic cell division were also identified. As for males, signaling-related pathways and others involved in the development of the nervous system were enriched. Altogether, these data point to a broad spectrum of alterations associated with the comorbidity model. Interactome maps, in turn, revealed a more restricted scenario. Nine of the eighteen deregulated proteins formed a network in females, and five of the fifteen deregulated proteins formed a network in males.

The nine proteins that composed the interactome map of females were grouped in interconnected subnetworks. One of these subnetworks contained three proteins of the TCA cycle [Citrate synthase (Cs), Aconitate hydratase_mitochondrial (Aco2), and Isocitrate dehydrogenase [NAD] subunit alpha_mitochondrial (Idh3a)]. All three proteins were downregulated in PCPNIC female mice, suggesting a reduction in energy production and associated metabolic dysfunction. These data are consistent with evidence for the role of aberrant energy metabolism in the etiology and pathogenesis of SCHZ [81,82]. An abnormal neuronal and glial energy state has broad implications. In addition to being the primary source of high-energy intermediates, mitochondria contribute to intracellular Ca^2+^ buffering, redox balance, and apoptosis modulation [83,84]. Accordingly, mitochondrial dysfunction may impair one or more of these functions. Given the brain’s high energy demands and the crucial role of Ca^2+^ in neuronal function, mitochondrial hypofunction may impair neurotransmission—an impairment possibly aggravated by imbalances in redox homeostasis and apoptosis regulation [83]. Interestingly, substantial evidence suggests interactions between mitochondrial defects and abnormal dopamine metabolism. Among other effects [85], functional impairment of mitochondria was shown to reduce DA catabolism, leading to increased dopamine and reactive oxygen species levels [84]. Among the downregulated TCA cycle proteins, CS, as a rate-limiting enzyme in the beginning of the TCA cycle, may have an important role in these outcomes. Of note, Cs was also shown to be deregulated in the medial prefrontal cortex of female mouse co-models of SCHZ and nicotine misuse [45], which points to a deregulation common to both the mesocortical and mesolimbic systems and suggests that Cs should be further investigated as a bioenergy synthesis and mitochondrial respiration biomarker for the comorbidity in females.

The second subnetwork included three downregulated proteins of the 14-3-3 family [C-terminal-binding protein 1 (Ctbp1), 14-3-3 protein gamma (Ywhag), and 14-3-3 protein epsilon (Ywhae)] and was connected to the third subnetwork, composed of proteins related to G protein-coupled receptor signaling [cAMP-dependent protein kinase catalytic subunit alpha (Prkaca), Guanine nucleotide-binding protein subunit beta-4 (Gnb4), and Sodium/potassium-transporting ATPase subunit alpha-2 (Atp1a2)]. 14-3-3 proteins, including the epsilon and gamma isoforms, are involved in several physiological processes such as cell proliferation and differentiation, autophagy, apoptosis, and cell cycle control [86,87]. Consistent with the aforementioned roles, here, the gamma isoform directly connected to Ctbp1, a transcriptional coregulator that is known to repress the expression of pro-apoptotic [88] and cell-cycle inhibitor genes [89]. Prkaca, the protein kinase A catalytic subunit, was connected not only to the Ywhag and Ywhae proteins but also to Gnb4 and Atp1a2. Considering that 14-3-3 proteins bind and modulate signaling proteins, including kinases [90,91], Ywhag and Ywhae downregulation could have a detrimental impact on Prkaca. Prkaca and Gnb4 are both components of G protein-mediated transmembrane signaling systems, while Atp1a2, as a subunit of the sodium/potassium-ATPase, indirectly contributes to neurotransmission balance. Considering the broad scope of cellular processes that are modulated by G protein signaling systems, the association between deficient G protein signaling and SCHZ needs further investigation. 14-3-3 family and G protein-coupled receptor subnetwork components were previously shown to be deregulated in SCHZ patients and in animal models of this disorder [92,93,94,95].

The protein–protein interaction network analysis of PCPNIC males included Mitogen-activated protein kinases 1 and 3 (Mapk1 and Mapk3), Actin alpha 1 (Acta1), ADP Ribosylation Factor 4 (Arf4), and Calcium/calmodulin-dependent protein kinase type II subunit alpha (Camk2a), which are proteins involved in cell signaling and associated mechanisms of cytoskeleton organization. Both Mapk1 and Mapk3 are part of the MAPK family, also known as extracellular signal-regulated kinases (ERKs). These kinases play crucial roles in regulating cellular processes such as proliferation and differentiation, memory/learning, and long-term potentiation in response to external stimuli. MAPKs are known to interact with microtubules and other cytoskeletal components [96,97], aligning with Mapk3 association with Acta1 in the male network. Acta1 is, in turn, linked to Arf4, a small GTPase predicted to regulate dendritic growth and vesicular trafficking [98]. Mapk3 is also connected to Camk2a, which is critical to several aspects of synaptic plasticity [99]. In addition to its role in the modulation of glutamatergic synapses, Camk2 can regulate the activity of proinflammatory proteins including MAPK [100], and its reduction is a common mechanism underlying social behavior and cognitive deficits associated with SCHZ [99]. Except for Arf4, the proteins Mapk1, Mapk3, and Acta1 were upregulated in the male interactome map, which may indicate that they play a role in cytoskeletal remodeling in response to external stimuli. Interestingly, increased Mapk3 expression and phosphorylation have been associated with increased dendritic arborization and the premature maturation of synapses in cortical pyramidal neurons in an animal model of 16p11.2 microduplication, a copy number variation strongly associated with SCHZ [101], possibly leading to the impaired formation of neuronal networks and associated cognitive deficits. A similar outcome was identified in a model of SCHZ-associated postsynaptic downregulation of miR-137, which causes accelerated maturation of excitatory synapses in hippocampal neurons, leading to their premature conversion from silent to active synapses [102]. A transcriptome-wide association study (TWAS) using data from a large SCHZ genome-wide association study (GWAS) identified a genetic correlation between Mapk3 expression and this mental disorder [103]. This protein was also deregulated in the medial prefrontal cortex of male mouse co-models of SCHZ and nicotine misuse [45], suggesting that Mapk3 could be of interest as a candidate marker of the comorbidity, associated with intracellular signaling, cytoskeleton reorganization, and synaptic plasticity in males.

### 4.2. Commonly Deregulated Proteins in NIC and PCPNIC Mice

Commonly deregulated proteins in NIC and PCPNIC adolescent females represent contributions of nicotine history to the NAcc profile of PCPNIC differentially expressed proteins. In females, 15 commonly deregulated proteins fit into the top Reactome pathways associated with the immune response, glucose metabolism, MAPK signaling, and cell death. Consistent with these findings, 12 of these proteins were present in the interactome map and grouped in two independent subnetworks. Fructose-bisphosphate aldolase A (Aldoa) and Pyruvate kinase (Pkm) are key enzymes in glycolysis, while L-lactate dehydrogenase B chain (Ldhb) is one of the subunits of lactate dehydrogenase, an enzyme that converts pyruvate into lactate. In the map, Aldoa is linked to Glutathione S-transferase P 1 (Gstp1), an oxidative stress protein. The downregulation of these proteins corroborates the well-known role of energy metabolism dysfunction and oxidative stress in SCHZ [82] and in tobacco smoking [104,105]. The T-complex protein 1 subunit epsilon (Cct5), a subunit of the T complex protein 1 (TCP-1), is the fifth protein of the first subnetwork. TCP-1 is a molecular chaperone that assists with the proper folding of proteins, including actin, in an ATP-dependent manner [106]. The map shows Cct5 connected to Ldhb, which implies that the energy metabolism imbalance might impact the folding of proteins. Further studies are needed to verify whether TCP-1 downregulation leads to the improper folding of actin, contributing to the cytoskeleton dysregulation in SCHZ patients [107]. TCP-1 was previously shown to be downregulated in SCHZ patients [107] and in rodents exposed to nicotine [108].

Microtubule-associated protein tau (Mapt), CaM kinase-like vesicle-associated protein (Camkv), Calcium/calmodulin-dependent protein kinase type II subunit gamma (Camk2g), 14-3-3 protein beta/alpha (Ywhab), Drebrin (Dbn1), Septin-7 (Sept7), and Ras-related protein Rab-8A (Rab8a) compose the second male subnetwork. Mapt stood out as centrally located, being connected to the two kinases (Camkv and Camk2g), Ywhab, and Dbn1, which suggests an important role of Mapt in cytoskeleton reorganization in NIC and PCPNIC females. The microtubule dynamics facilitates neurodevelopmental processes including axon elongation, dendrite arborization, and synapse formation. Moreover, microtubules further contribute to the maintenance of axon and dendrite structure and to intracellular trafficking throughout life [109]. Mapt plays a key role in stabilizing microtubules; accordingly, its downregulation has the potential to cause serious negative consequences. In this regard, Mapt inhibition leads to microtubule decomposition, an initial step of dendritic pruning [110]. This effect is consistent with the excessive dendritic pruning identified in high-risk youth and early-onset SCHZ patients [111]. Mapt is downregulated in SCHZ patients and has been described as a risk gene for this disorder [112,113]. Nicotine and other modulators of nicotinic cholinergic receptors were also shown to affect Mapt. However, most studies focus on Mapt phosphorylation levels rather than its expression, and both reductions [114] and increases [115] were described. Mapt connections to Camk2g and Camkv point to a regulatory role of these kinases in microtubule dynamics. In accordance with this possibility, CaMKv is a pseudokinase essential for dendritic spine maintenance and neuroplasticity [116]. Similarly, Camk2g is well known for its role in neurological processes such as synaptic plasticity and memory [117], and it is a key kinase responsible for Mapt phosphorylation [118,119]. In the interactome map, Camk2g is also directly linked to Ywhab, which aligns with evidence of 14-3-3 protein interactions with kinases and their role as regulators of intracellular signaling [91]. Camk2 is a point of convergence of signaling pathways associated with SCHZ [120], and its activity is increased in brain reward regions such as the NAcc in response to nicotine exposure [121]. As described above, Camk2a, another isoform of Camk2, is one of the exclusively deregulated proteins in PCPNIC males, so that the identification of the deregulation of other related kinases in PCPNIC females strengthens the importance of the investigation of the role of isoforms of this protein in the comorbidity. Camk2 regulates MAPK [100], which, in turn, interacts with the microtubule cytoskeleton [96,97]. Finally, Mapt connects with Dbn1, a regulator of the actin cytoskeleton in dendritic spines [122]. Dbn1 also plays a role in the coordination of the cross-talk between actin and microtubules, which is important for neuritogenesis [122]. Dbn1, in turn, is linked to Sept7, which is linked to Rab8a. Sept7 is a member of the septin cytoskeleton that promotes dendritic spine formation and contributes to spine stability and maturation by interacting with other cytoskeleton components, including microtubules [123]. As for Rab8a, it is a component of the Rab GTPase family that, among other roles, participates in autophagy processes [124,125]. In particular, Rab8 is required for secretory autophagy, a trans-synaptic signaling mechanism important for activity-induced synaptic remodeling [126]. Except for Camk2g, the other proteins that compose the cytoskeleton dynamics subnetwork were downregulated, consistent with evidence of reduced synaptic plasticity in SCHZ [127] and the proposed role of the altered cytoskeleton dynamics in substance use disorders [128].

In males, three deregulated proteins in NIC mice contributed to the NAcc profile of PCPNIC differentially expressed proteins. Those were associated with transport and binding, protein SUMOylation, and lipid metabolism Reactome pathways. Beta-adducin (Add2) and Heterogeneous nuclear ribonucleoprotein K (Hnrnpk) were downregulated in both the NIC and PCPNIC groups. Add2 is an adducin family protein highly expressed in dendritic spines and is involved in the remodeling of the actin cytoskeleton and associated assembly of new synapses, events required for memory and learning [129,130]. Consistent with this finding, the investigation of Add2 polymorphisms suggests that Add2 is involved in cognitive impairment in SCHZ [131]. Hnrnpk is a DNA/RNA-binding protein known to integrate signal transduction pathways with gene expression by participating in a variety of events such as chromatin remodeling, transcription, RNA splicing, and translation [132]. Even though heterogeneous nuclear ribonucleoprotein C, another member of the family, was reported to be downregulated in the brain of SCHZ patients [133], this is the first study that reports Hnrnpk deregulation in association with nicotine misuse and SCHZ. Distinct from Add2 and Hnrnpk, Prosaposin (Psap), the third commonly deregulated protein in the NAcc of NIC and PCPNIC males, was upregulated. Psap is a modulator of glycosphingolipid metabolism and also a neurotrophic factor [134,135]. In particular, it was shown to maintain lipid homeostasis in dopaminergic neurons of rodents [136] and to promote cell survival and neurite outgrowth in a cholinergic cell line [135]. Accordingly, its upregulation might play a role in the reported positive effects of nicotine on SCHZ patients [39] and animal models [42,43]. This is the first study that reports that Psap exhibited altered expression in the NAcc in a SCHZ + nicotine misuse comorbid model. Of note, nucleotide polymorphisms in Psap were found to be associated with SCHZ [137], and sphingolipid metabolism is altered in SCHZ patients [138].

### 4.3. Commonly Deregulated Proteins in PCP and PCPNIC Mice

Commonly deregulated proteins in PCP and PCPNIC adolescent mice represent contributions of phencyclidine to the NAcc profile of PCPNIC differentially expressed proteins. Among the top 10 enriched Reactome pathways, nervous system development was common to both sexes, and Neural cell adhesion molecule 1 (Ncam1) was commonly downregulated in PCP and PCPNIC females and males. Ncam1 is a membrane glycoprotein that regulates cell-to-cell adhesion and neurite growth, ultimately modulating the strength and morphology of synaptic connections [139]. When injected in the cerebrospinal fluid of mice, Ncam1 antibodies inhibit trans-homophilic (NCAM1-NCAM1) binding and NCAM1 binding to glial-cell-line-derived neurotrophic factor, leading to a reduction in the number of spines and synapses in the frontal cortex [140]. In case a similar effect occurs in our model, the Ncam1 downregulation could exacerbate synaptic pruning, resulting in the impaired maturation of neural circuits relevant to SCHZ [127]. Ncam1 was also shown to modulate the development of GABAergic synapses [141], suggesting that its disbalance negatively impacts distinct neurotransmitter systems. Several pieces of evidence link Ncam1 and SCHZ: first-episode drug-naïve SCHZ patients show decreased serum Ncam levels, and this protein’s levels positively correlate with cognitive scores [142]; and single nucleotide polymorphisms of the Ncam1 gene are associated with cognitive deficiencies in SCHZ patients [143]. The decreased expression of this protein was identified in neural progenitor cells derived from SCHZ patients [144], suggesting that Ncam1 may play a role in the initiation of the disease. Interestingly, Ncam1 autoantibodies are present in subpopulations of SCHZ patients, and when injected in the cerebrospinal fluid of mice, these antibodies induce SCHZ-related behaviors [140]. Ncam1 downregulation in both males and females suggests that this protein disbalance lies at the core of SCHZ pathophysiology. In accordance with these findings, Ncam1 has been proposed as a marker of synaptic plasticity in SCHZ [139].

While in males, Ncam1 was the sole commonly deregulated protein, in females, the Reactome analysis of the 17 commonly deregulated proteins identified pathways related to the immune system, metabolism of proteins, signal transduction, vesicle-mediated transport, and membrane trafficking as the most frequent ones. As expected, the interactome map revealed a more restricted scenario. Seven of the seventeen deregulated proteins were grouped into three independent subnetworks. In the first subnetwork, Synaptotagmin-1 (Syt1) was centrally located and connected to Ncam1 and Ras-related protein Rab-3D (Rab3d). Syt1 is a Ca^2+^-dependent sensor of synaptic vesicle release [145,146] that, recently, was proposed to have bidirectional and opposite roles in exocytosis–endocytosis by facilitating clathrin-mediated endocytosis but inhibiting bulk endocytosis [147]. Syt1 was upregulated in the PCP group, which is consistent with previous finding in SCHZ patients [148]. However, it was downregulated in PCPNIC mice, which suggests that the effect of phencyclidine on the expression of Syt1 in the NAcc of PCPNIC mice was significantly altered by nicotine history. The other two proteins of the subnetwork (Ncam1 and Rab3d) were downregulated in both PCP and PCPNIC females. Rab proteins belong to the Rab family of the small GTPase superfamily. Rab3 isoforms, including Rab3d, are highly expressed in synaptic vesicles [67], playing roles in several stages of vesicular transport and membrane fusion [149] and regulating vesicular release probability [150]. To our knowledge, this is the first report of an association between Rab3d and SCHZ. The Ncam1 connection to Syt1 in the interactome map of females may be due to its role in modulating synaptic connections [139].

As for the second subnetwork, the downregulation of Actin-related protein 2/3 complex subunit 4 (Arpc4) and Actin_ alpha cardiac muscle (Actc1) in PCP and PCPNIC female mice contributes to the perception that a dysfunctional cytoskeleton plays a role in the remodeling of axon terminals and dendritic arbors that underlies alterations in synaptic connectivity in SCHZ patients [151]. Arpc4 is a subunit of the actin-related protein 2/3 (Arp2/3) complex, a key component of the actin cytoskeleton that acts as a nucleation site for the formation and polymerization of new filaments of actin, a process that is essential for functions such as phagocytosis and vesicular trafficking [152]. In neurons, the Arp2/3 complex also plays a crucial role in dendritic spine morphogenesis, aiding in the maturation of the dendritic spine phenotype [153]. Arpc4 is downregulated in cortical regions relevant to visual working memory in SCHZ subjects [154]. To our knowledge, this is the first study that shows its downregulation in the NAcc in an animal model. Arpc4 is linked to Actc1. Actc1 is primarily expressed in cardiac muscle cells, and even though it has also been identified in the brain (https://www.proteinatlas.org/ENSG00000159251-ACTC1/brain, accessed on 6 January, 2025), a few studies focus on its function in neurons and/or glial cells. It was shown to be expressed during the neural differentiation of murine embryonic stem cells exposed to the neurotoxicant valproic acid [155] and in glioblastoma cells [156]. Future studies are warranted to verify the impact of Actc1 downregulation in SCHZ and in the SCHZ + nicotine misuse comorbidity.

The last subnetwork of proteins commonly deregulated in PCP and PCPNIC adolescent female mice comprises two proteins that play important roles in translation, a critical and highly energetically demanding stage of the gene expression process [157]. Eukaryotic initiation factor 4A-II (Eif4a2) is the enzymatic core of the eukaryotic initiation factor 4F complex. It is an ATP-dependent RNA helicase that helps with cap recognition and mRNA binding to ribosomes, making it essential for translation initiation, the rate-limiting step in the synthesis of most proteins [157]. Eukaryotic translation elongation factor 1 gamma (Eef1g), participates in the second phase of translation, named elongation, as a component of the eukaryotic elongation factor-1 complex, which binds to the aminoacyl-tRNAs and transfers them to the ribosome [158]. Eif4a2 was recently proposed to be a hub gene out of 17 signature genes of SCHZ [159]. However, Eef1g is a new candidate gene. To our knowledge, this is the first study that shows Eef1g deregulation in animal models of SCHZ and SCHZ + nicotine misuse comorbidity. Both Eif4a2 and Eef1g were downregulated in PCP and PCPNIC females. If similar outcomes occur in SCHZ patients, those could contribute to the decreased levels of various classes of proteins that occur in this mental disorder.

### 4.4. Commonly Deregulated Proteins in PCP, NIC, and PCPNIC Mice

Epidemiological, clinical, and biological data support the notion that the dysfunction of the immune system has a central role in the pathophysiology of SCHZ [160]. Even though the underlying causes remain unknown, SCHZ patients frequently exhibit characteristics of immune disorders, including prior infections [161], the presence of autoantibodies [162], comorbid autoimmunity diseases [163], and inflammation [164]. Consistent with the immune hypothesis of SCHZ, in females, the common contributions of PCP and NIC to the PCPNIC deregulated profile included 10 proteins mostly associated with interleukin signaling. The map for proteins deregulated in all three drug-exposed groups was composed of one network that included 2 of the 10 deregulated proteins.

Complement C1q Binding Protein (C1qbp) binds C1q, a classic immune mediator of the complement pathway that has a well-established role in antigen elimination [165]. Interestingly, a role of Cq1 in synapse maturation and plasticity modulation has also been reported. Cq1, along with other complement proteins, has been suggested to tag synapses for subsequent elimination, thus being proposed as a key component in synaptic pruning [166]. Considering that C1q seropositivity has been associated with SCHZ in recent-onset patients [167], the possibility that the upregulation of C1qbp plays a role in the excessive dendritic pruning identified in SCHZ patients [111] is worth further investigation.

C1qbp is connected to 60 kDa heat shock protein_ mitochondrial (Hspd1). Hspd1 has been associated with SCHZ risk [168]. It is a chaperone protein that maintains protein homeostasis, including protein folding, unfolding, and disaggregation [169]. However, in addition to these canonical roles, Hspd1 participates in the inflammatory response [170], has a protective role in neurodegenerative diseases [171], and regulates myelination [172]. It has been suggested that immune genes implicated in SCHZ have dual roles in immunity and central nervous system function [173]. Here, both C1qbp and Hspd1 fit this profile, which raises the possibility that these proteins are a link between the immune system and this mental disorder. The immune pathways involved in SCHZ remain under investigation. However, while we failed to find common contributions of the insults to the comorbid model in males, the proteins identified in females are immune-related, which suggests that the role of the immune perturbations in SCHZ may be particularity relevant in females.

## 5. Conclusions

In this study, we preclinically identified proteins that are differentially expressed exclusively in PCPNIC mice, revealing unique effects associated with the comorbidity SCHZ + nicotine history model. Among potential early candidate markers of the combined insult in the NAcc, Cs and Mapk3 stood out in females and males, respectively. Both proteins were previously shown to be exclusively deregulated in the medial prefrontal cortex of mouse co-models of SCHZ and nicotine misuse [45], which points to a deregulation common to both the mesocortical and mesolimbic systems. The deregulation of these and other connected proteins in the NAcc interactome maps is consistent with evidence for the role of aberrant energy metabolism and synaptic plasticity in our comorbidity model.

Our experimental design further allowed for the evaluation of the impact of nicotine history in mouse models of SCHZ and the contributions of the SCHZ model to the profile of deregulated proteins in mouse models of the comorbid condition. Regarding the impact of nicotine, Mapt was centrally located in a subnetwork associated with the cytoskeleton dynamics in females, pointing to a role of the cytoskeleton in the pathophysiology of the comorbidity. As for the contributions of the SCHZ model, Ncam1 could be of interest as a candidate marker of synaptic plasticity disbalance. Its deregulation in the NAcc of both males and females suggests that Ncam1 lies at the core of the comorbidity pathophysiology.

A few new comorbidity candidates were identified—proteins that had not previously been reported as dysregulated in preclinical or clinical studies involving SCHZ and nicotine/tobacco use. Rab3d plays roles in several stages of vesicular transport and membrane fusion, and Eef1g participates in the elongation phase of translation. Both were commonly deregulated in PCP and PCPNIC females, and their identification could potentially offer insights into novel mechanisms underlying the co-occurrence of SCHZ and nicotine misuse.

Sex differences in SCHZ and nicotine misuse are well known. A limitation of the current study is that the methodological design does not allow for the investigation of whether genetic, hormonal, or interactions between these factors are responsible for the sex-selective changes in protein expression. Despite that, the larger number of deregulated proteins in females, a result that was also identified in a previous study that investigated the medial prefrontal cortex [45], as well as the distinct profile of proteins deregulated in males vs. females suggest that all three possibilities are involved in explaining our results, albeit in a protein-dependent way. Another limitation is the use of NAcc tissue homogenates, which prevents the identification of the specific cell type(s) involved in protein deregulation, thereby limiting a deeper understanding of the data. Future studies employing single-cell RNA sequencing or mass spectrometry analysis of cell cultures are warranted and would further increase our knowledge of schizophrenia and nicotine misuse comorbidity. Lastly, the current data do not allow us to infer whether the protein deregulations identified are downstream factors in the comorbid pathophysiology and, as such, modifiers of the disorder outcomes; whether they are byproducts; or whether they have a causal role in the comorbidity. Notwithstanding, here, we provide relevant information concerning potential pathological markers of the combined phencyclidine and nicotine insults during adolescence. Our results highlight that, during adolescence, an important period for both nicotine misuse and SCHZ establishment, the comorbid condition might comprise protein imbalances that are, to a great extent, unique when compared to SCHZ and nicotine misuse disorders, and that, as such, need to be further investigated. In this sense, the current study has the potential to direct future studies aiming to identify the mechanisms of SCHZ + nicotine misuse in the early course of this comorbidity, a time window that is critical for early intervention.

## Data Availability

All raw data are available as Supplementary Materials.

## Supplementary Materials

The following supporting information can be downloaded at: https://www.mdpi.com/article/10.3390/biomedicines13040901/s1, Supplementary Material S1: Methods used to establish the normality and significance of data; Supplementary Material S2: Other additional data; Supplementary Material S3: Data from deregulated proteins in the NAcc of female and male mice.

## Abbreviations

The following abbreviations are used in this manuscript:

ACO2	Aconitate hydratase_ mitochondrial
Acta1	Actin alpha 1
Actc1	Actin_ alpha cardiac muscle
Add2	Beta-adducin
Aldoa	Fructose-bisphosphate aldolase A
Arf	ADP Ribosylation Factor 4
Arpc4	Actin-related protein 2/3 complex subunit 4
Atp1a2	Sodium/potassium-transporting ATPase subunit alpha-2
C1qbp	Complement C1q Binding Protein
Camk2a	Calcium/calmodulin-dependent protein kinase type II subunit alpha
Camk2g	Calcium/calmodulin-dependent protein kinase type II subunit gamma
Camkv	CaM kinase-like vesicle-associated protein
Cct5	T-complex protein 1 subunit epsilon
CS	Citrate synthase
CT	Control group
Ctbp1	C-terminal-binding protein 1
DIA	Data-independent acquisition
Dbn1	Drebrin
DTT	Dithiothreitol
Eef1g	Eukaryotic translation elongation factor 1 gamma
Eif4a2	Eukaryotic initiation factor 4A-II
ENDS	Electronic nicotine delivery system
FDR	False discovery rate
GFP	[Glu1]-Fibrinopeptide B human
Gnb4	Guanine nucleotide-binding protein subunit beta-4
Gstp1	Glutathione S-transferase P 1
GWAS	Genome-wide association study
Hnrnpk	Heterogeneous nuclear ribonucleoprotein K
Hspd1	60 kDa heat shock protein_mitochondrial
IAA	Iodoacetamide
Idh3a	Isocitrate dehydrogenase [NAD] subunit alpha_ mitochondrial
Ldhb	L-lactate dehydrogenase B chain
Mapk1	Mitogen-activated protein kinase 1
Mapk3	Mitogen-activated protein kinase 3
Mapt	Microtubule-associated protein tau
MSE	Mass spectrometry Experimentation
NAcc	Nucleus Accumbens
Ncam1	Neural cell adhesion molecule 1
NIC	Nicotine-exposed group
PANTHER	Protein Analysis Through Evolutionary Relationships
PCP	Phencyclidine-exposed group
PCPNIC	Phencyclidine- and nicotine-exposed group
PN	Postnatal day
Prkaca	cAMP-dependent protein kinase catalytic subunit alpha
Psap	Prosaposin
Rab3d	Ras-related protein Rab-3D
Rab8a	Ras-related protein Rab-8A
SCHZ	Schizophrenia
Sept7	Septin-7
Syt1	Synaptotagmin-1
TCA	Tricarboxylic acid cycle, also known as the citric acid cycle and Krebs cycle
TEAB	Triethylammonium bicarbonate buffer
TWAS	transcriptome-wide association study
VTA	Ventral tegmental area
Ywhab	14-3-3 protein beta/alpha
Ywhae	14-3-3 protein epsilon (Ywhae)
Ywhag	14-3-3 protein gamma
