# Supplementary material for: Nucleus Accumbens Proteome Disbalance in an Adolescent Mouse Model of Schizophrenia and Nicotine Misuse Comorbidity
Source: Biomedicines. 2025 Apr 8;13(4):901. doi: 10.3390/biomedicines13040901 (PMC12025060; doi:10.3390/biomedicines13040901)

SUPPLEMENTARY MATERIAL 1

Biomedicines

Nucleus Accumbens proteome disbalance in an adolescent mice model of schizophrenia and nicotine misuse comorbidity

Thainá Pereira de Souza[1], Andrés Rodríguez-Vega[1], Ana Carolina Dutra-Tavares[2], Keila A. Semeão[1], Claudio Carneiro Filgueiras[1], Anderson Ribeiro-Carvalho[3], Alex Christian Manhães[1], Yael Abreu-Villaça[1*]

1. Laboratório de Neurofisiologia, Departamento de Ciências Fisiológicas, Instituto de Biologia Roberto Alcantara Gomes, Universidade do Estado do Rio de Janeiro (UERJ), Av. Prof. Manuel de Abreu 444, 5 andar – Vila Isabel, Rio de Janeiro, RJ, 20550-170, Brazil.
2. Departamento de Ciências Biomédicas e Saúde, Instituto de Biologia Roberto Alcantara Gomes, Universidade do Estado do Rio de Janeiro (UERJ), Cabo Frio 28905-320, RJ, Brazil.
3. Departamento de Ciências, Faculdade de Formação de Professores da Universidade do Estado do Rio de Janeiro, São Gonçalo, RJ, 24435-005, Brazil.

Corresponding author: Dr. Yael Abreu-Villaça
E–mail: yael_a_v@yahoo.com.br
yael.villaca@uerj.br
ORCID: 0000-0002-9801-6179

Short title: Adolescent nicotine and schizophrenia NAcc proteomic profile

Methods used to establish the normality and significance of data

Our study consists of a first approximation of results obtained experimentally using quantitative proteomics applied to SCHZ and nicotine misuse models. To establish the normality and significance of these proteomic data, we used Progenesis for proteomics, which is widely used for processing experimental proteomic data using a Hi-3 approach as described in Silva et al. [68]. The abundance of each peptide was calculated from its precursor ions and for each protein its abundance was calculated from the 3 most abundant peptides present in each of the replicates of all groups. The alignment as well as normalization were performed by the program for all runs and experiments, performing the average reading to calculate the relative quantification of the same protein in all runs. Examples of the normalization control performed by Progenesis are found below.

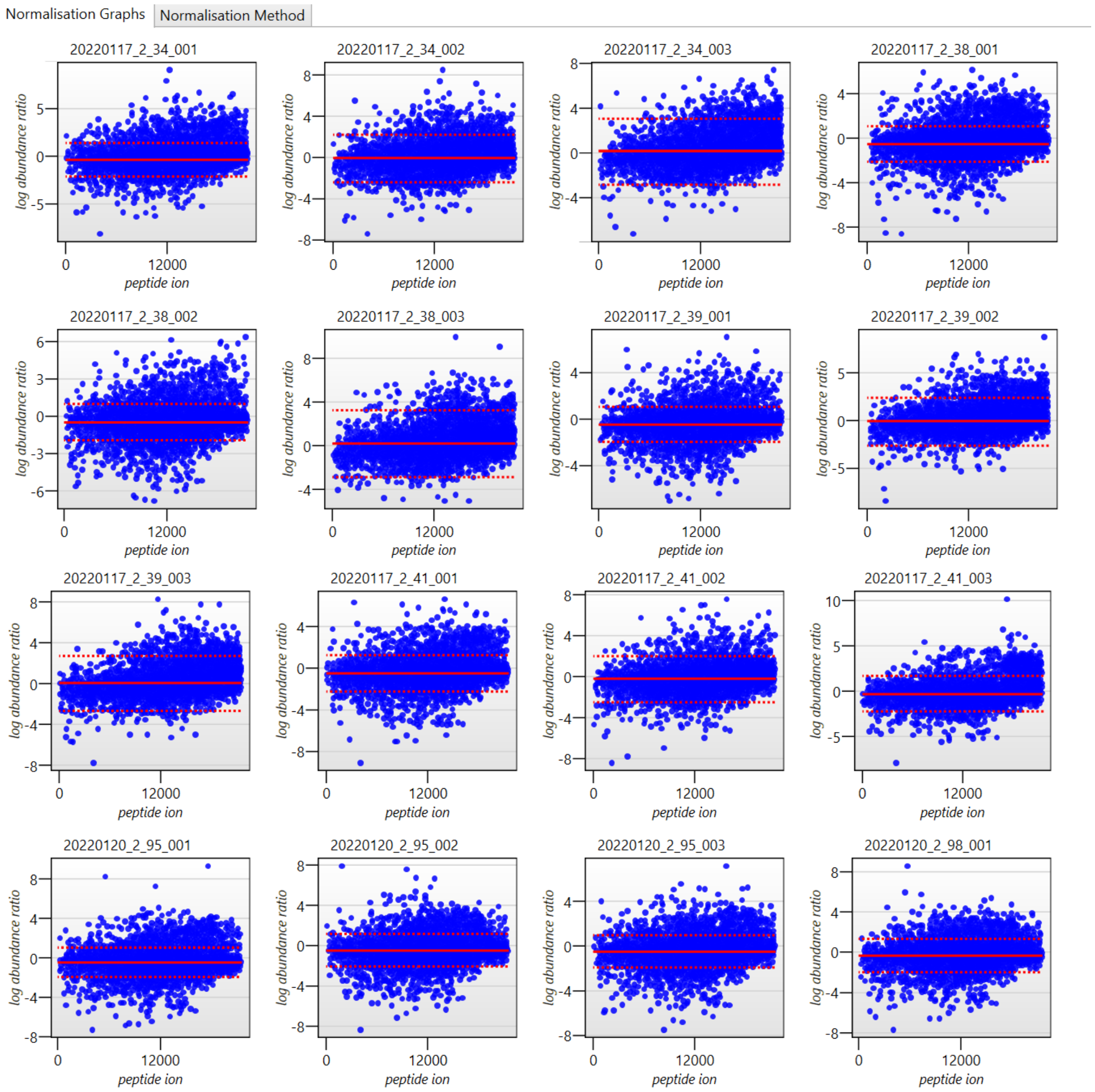

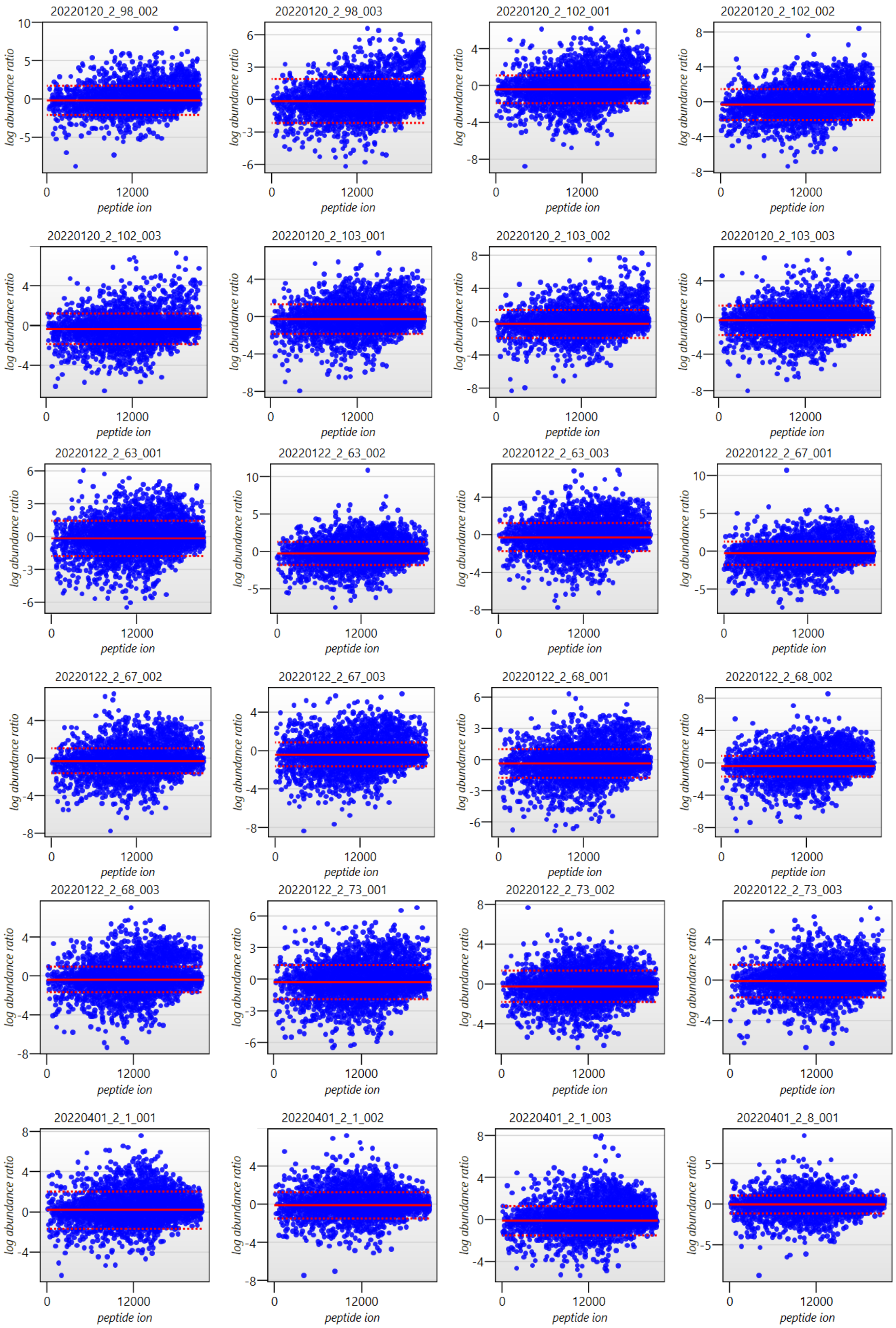
20220120_2_98_002
20220120_2_98_003
20220120_2_102_001
20220120_2_102_002
20220120_2_102_003
20220120_2_103_001
20220120_2_103_002
20220120_2_103_003
20220122_2_63_001
20220122_2_63_002
20220122_2_63_003
20220122_2_67_001
20220122_2_67_002
20220122_2_67_003
20220122_2_68_001
20220122_2_68_002
20220122_2_68_003
20220122_2_73_001
20220122_2_73_002
20220122_2_73_003
20220401_2_1_001
20220401_2_1_002
20220401_2_1_003
20220401_2_8_001
log abundance ratio
peptide ion
0
12000

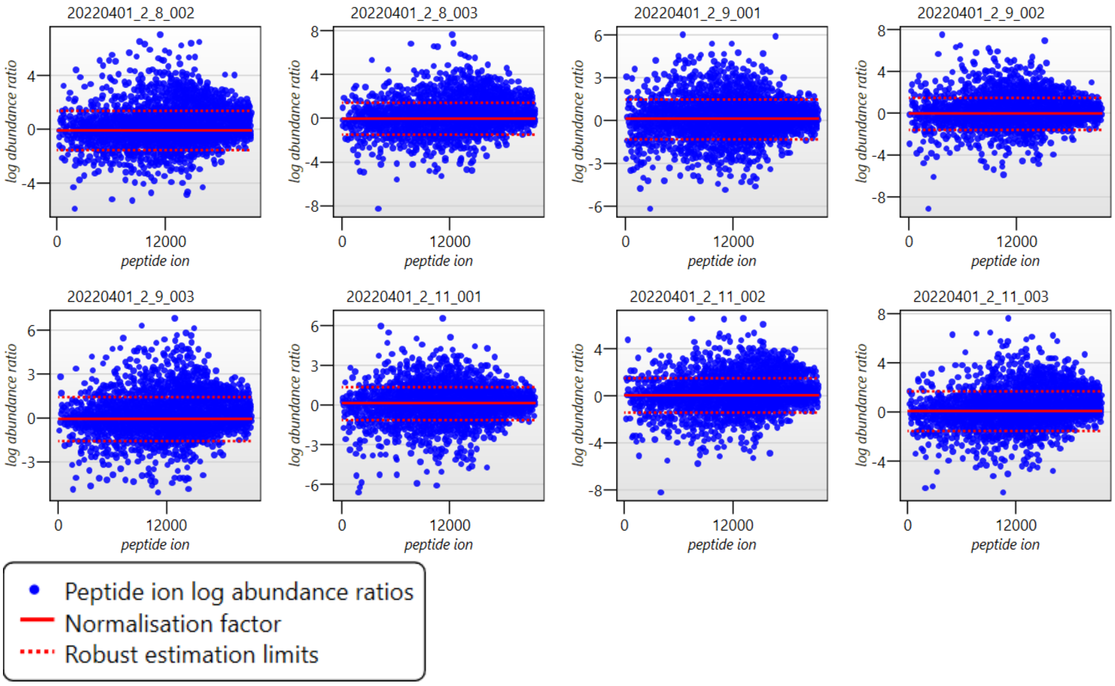

For each run, normalizations were performed for each peptide ion and only data within the robust limit calculated by Progenesis for the quantification of the presented proteins were considered. Data outside this limit were only considered and used to increase the weight of the identifications obtained in the proteomic experiment.

Other normalization graphs for the treatment of proteomic data in obtaining quantified proteins and peptides are presented below. These graphs show the number of peptides and proteins identified in each pairwise comparison run. Runs are grouped by experimental condition and the mean as well as the deviation of +/- 1 SD (Standard Deviation) are exemplified in each condition. Only peptides belonging to quantifiable proteins were considered for analysis.

CT *vs.* NIC Male proteins

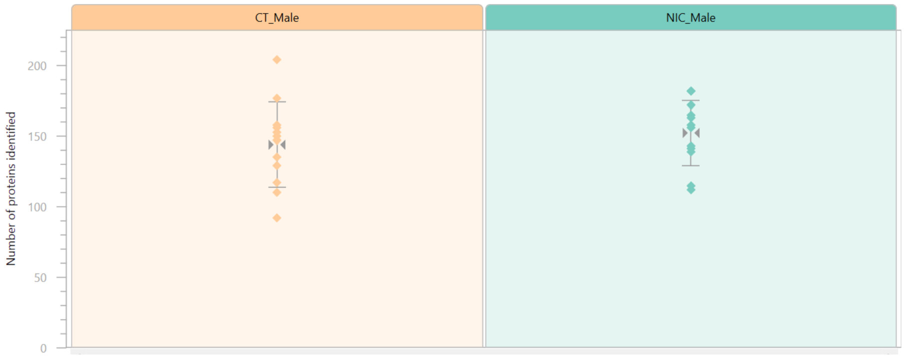

CT *vs.* NIC Male Peptides

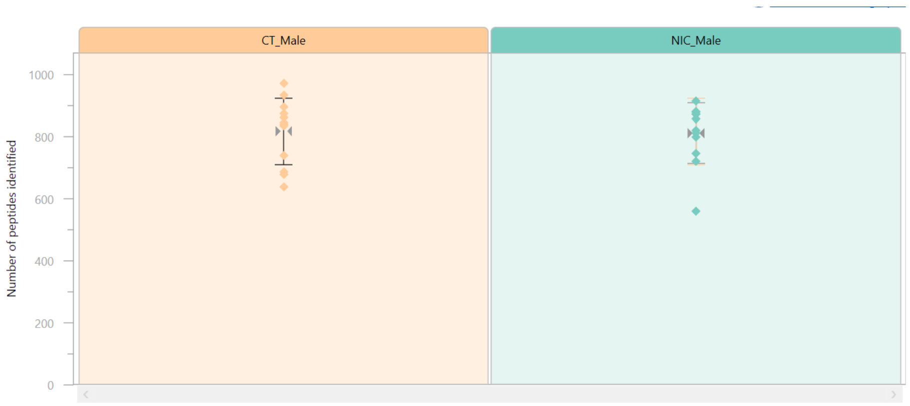

CT *vs.* PCPNIC Male Proteins

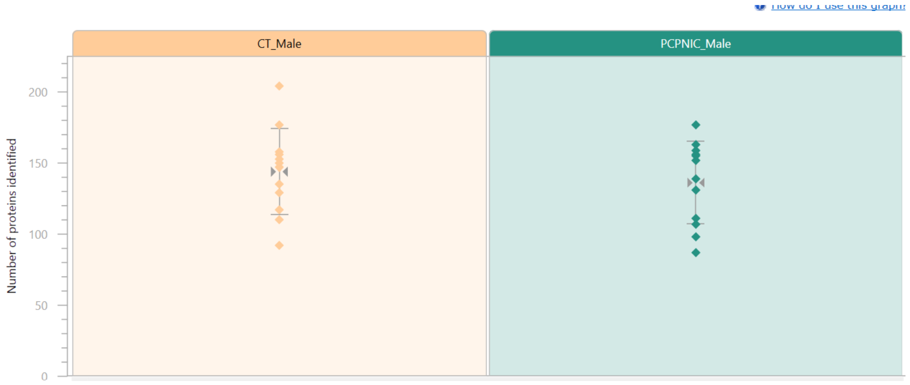

CT *vs.* PCPNIC Male Peptides

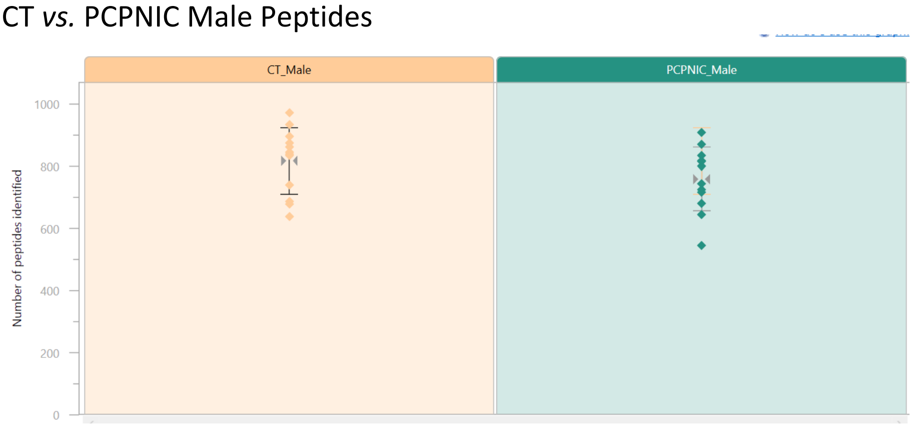

CT *vs.* PCP Male Proteins

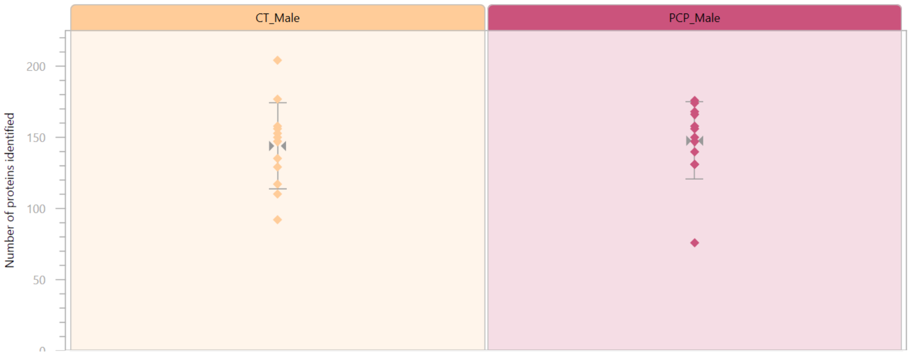

CT *vs.* PCP Males Peptides

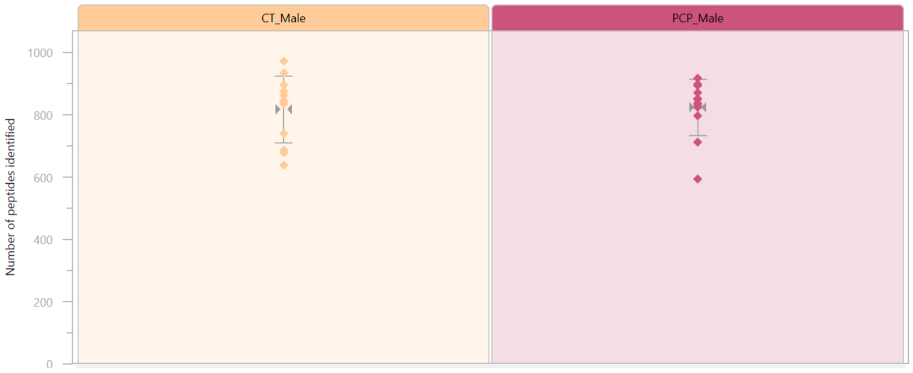

CT *vs.* NIC Female Proteins

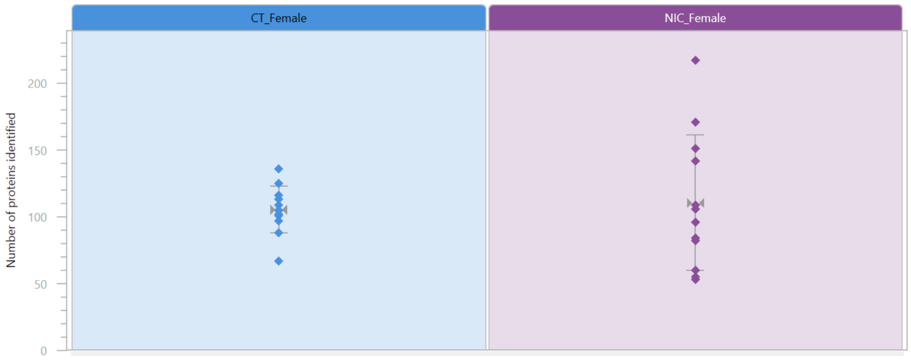

CT *vs.* NIC Female Peptides

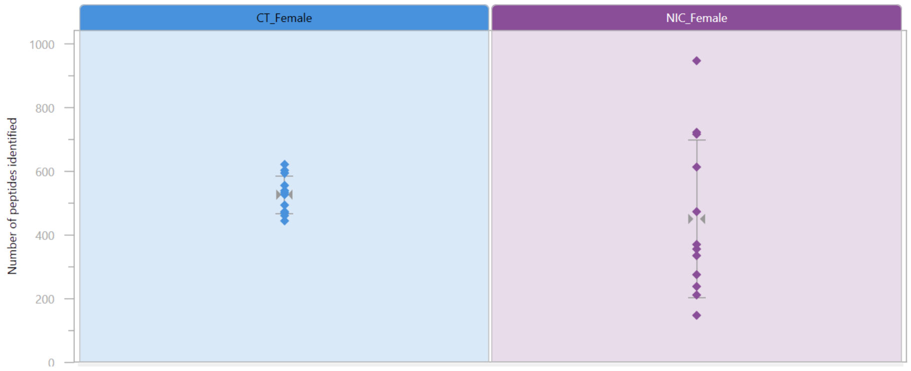

CT *vs.* PCPNIC Female Proteins

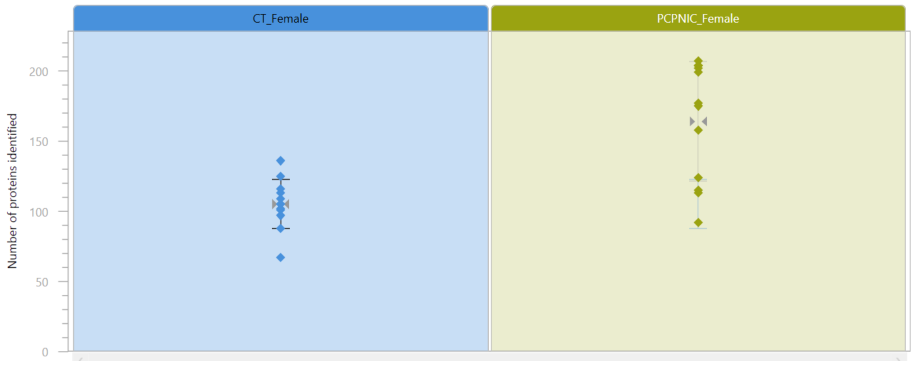

CT *vs.* PCPNIC Female Peptides

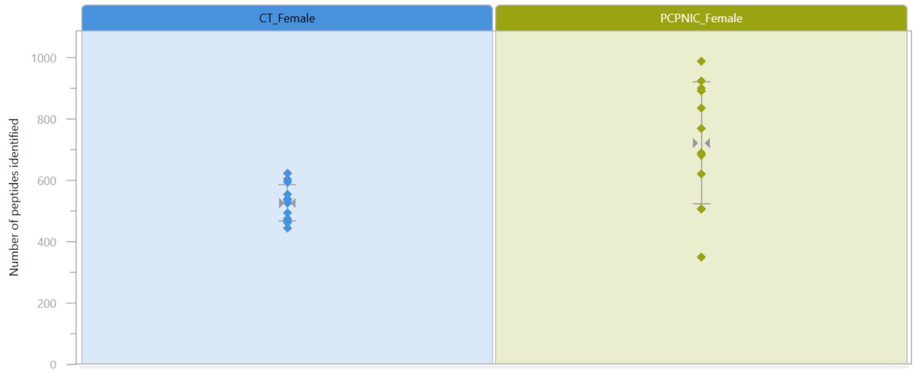

CT *vs.* PCP Female Proteins

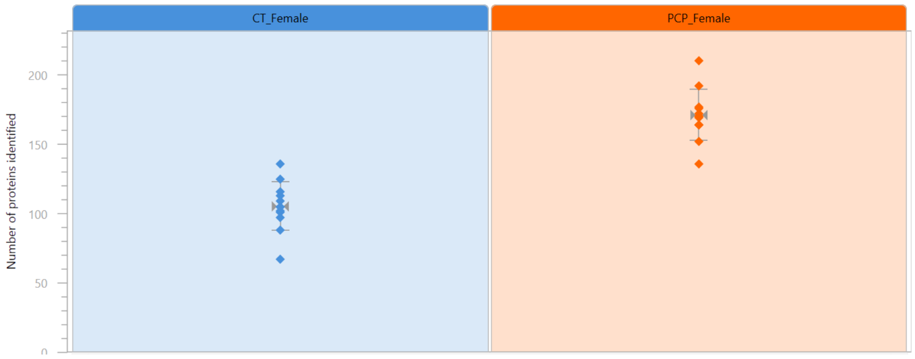

CT *vs.* PCP Female Peptides

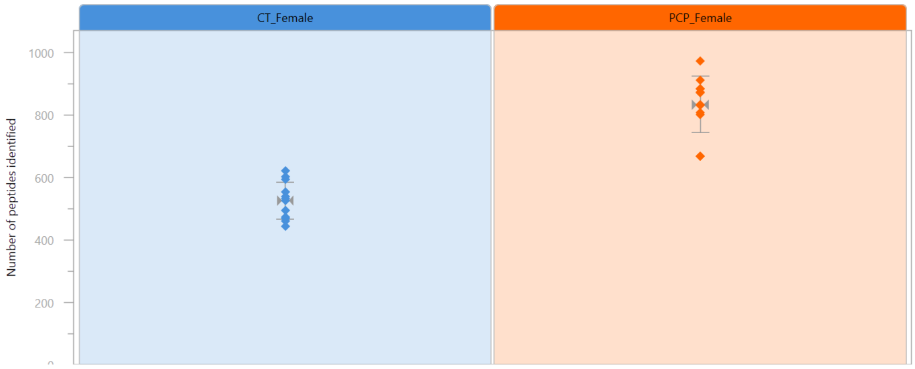

Supplement: Supplementary file 1 [file biomedicines-13-00901-s001.zip › biomedicines-3517699 Souza et al 2025 Supplementary Material S1 - R2.pdf]
