# Supplementary material for: Nucleus Accumbens Proteome Disbalance in an Adolescent Mouse Model of Schizophrenia and Nicotine Misuse Comorbidity
Source: Biomedicines. 2025 Apr 8;13(4):901. doi: 10.3390/biomedicines13040901 (PMC12025060; doi:10.3390/biomedicines13040901)
Supplement: Supplementary file 1 [file biomedicines-13-00901-s001.zip › biomedicines-3517699 Souza et al 2025 Supplementary Material S3 - Figures R2.pdf]

SUPPLEMENTARY MATERIAL 3

Biomedicines

Nucleus Accumbens proteome disbalance in an adolescent mice model of schizophrenia and nicotine misuse comorbidity

Thainá Pereira de Souza[1], Andrés Rodríguez-Vega[1], Ana Carolina Dutra-Tavares[2], Keila A. Semeão[1], Claudio Carneiro Filgueiras[1], Anderson Ribeiro-Carvalho[3], Alex Christian Manhães[1], Yael Abreu-Villaça[1*]

1. Laboratório de Neurofisiologia, Departamento de Ciências Fisiológicas, Instituto de Biologia Roberto Alcantara Gomes, Universidade do Estado do Rio de Janeiro (UERJ), Av. Prof. Manuel de Abreu 444, 5 andar – Vila Isabel, Rio de Janeiro, RJ, 20550-170, Brazil.

2. Departamento de Ciências Biomédicas e Saúde, Instituto de Biologia Roberto Alcantara Gomes, Universidade do Estado do Rio de Janeiro (UERJ), Cabo Frio 28905-320, RJ, Brazil.

3. Departamento de Ciências, Faculdade de Formação de Professores da Universidade do Estado do Rio de Janeiro, São Gonçalo, RJ, 24435-005, Brazil.

Corresponding author: Dr. Yael Abreu-Villaça
E–mail: yael_a_v@yahoo.com.br
yael.villaca@uerj.br
ORCID: 0000-0002-9801-6179

Short title: Adolescent nicotine and schizophrenia NAcc proteomic profile

Supplementary figures 1 to 4. Data from deregulated proteins in the NAcc of female and male mice. **Fig 1a to 1k**: Exclusively deregulated in mice modeled to the comorbidity (PCPNIC) when compared to controls (CT). **Fig 2a to 2d**: Commonly deregulated in mice modeled to nicotine misuse (NIC) and the comorbidity (PCPNIC), when compared to CT. **Fig 3a to 3c.** Commonly deregulated in mice modeled to schizophrenia (PCP) and the comorbidity (PCPNIC), when compared to CT . **Fig 4a to 4b.** Commonly deregulated in all experimental groups when compared to CT. The panels show complete lists of Reactome pathways. Pathways in bold and underlined were shared between sexes. ▼ indicates that a given protein is downregulated in a given comparison, while ▲ indicates that a given protein is upregulated.

Exclusively deregulated in PCPNIC *vs.* CT

Aco2: Aconitate hydratase_ mitochondrial; **Acta1**: Actin_ alpha skeletal muscle; **Amph**: Amphiphysin; **Arf4**: ADP-ribosylation factor 4; **Arpc1a**: Actin-related protein 2/3 complex subunit 1A; **Atp1a2**: Sodium/potassium-transporting ATPase subunit alpha-2; **Camk2a**: Calcium/calmodulin-dependent protein kinase type II subunit alpha; **Cbr1**: Carbonyl reductase [NADPH] 1; **Cfap20**: Cilia- and flagella-associated protein 20; **Col6a6**: Collagen alpha-6(VI) chain; **Cs**: Citrate synthase_ mitochondrial; **Ctbp1**: C-terminal-binding protein 1; **Eif4a2**: Eukaryotic initiation factor 4A-II; **Gda**: Guanine deaminase; **Gnb4**: Guanine nucleotide-binding protein subunit beta-4; **Hdhd2**: Haloacid dehalogenase-like hydrolase domain-containing protein 2; **Hspa2**: Heat shock-related 70 kDa protein 2; **Idh3a**: Isocitrate dehydrogenase [NAD] subunit alpha_ mitochondrial; **Mapk1**: Mitogen-activated protein kinase 1; **Mapk3**: Mitogen-activated protein kinase 3; **Ndrg2**: Protein NDRG2; **Phf24**: PHD finger protein 24; **Ppp1r7**: Protein phosphatase 1 regulatory subunit 7; **Prkaca**: cAMP-dependent protein kinase catalytic subunit alpha; **Rab4b**: Ras-related protein Rab-4B; **Sh3gl2**: Endophilin-A1; **Sntb1**: Beta-1-syntrophin; **Stx1a**: Syntaxin-1A; **Tppp**: Tubulin polymerization-promoting protein; **Uchl1**: Ubiquitin carboxyl-terminal hydrolase isozyme L1; **Ywhae**: 14-3-3 protein epsilon; **Ywhag**: 14-3-3 protein gamma.

REACTOME PATHWAYS	# OF PTNS	GENES												
**FEMALES**		Aco2	Atp1a2	Cs	Ctbp1	Gda	Gnb4	Hspa2	Idh3a	Prkaca	Sh3gl2	Rab4b	Ywhae	Ywhag
REGULATION: PCPNIC *vs.* CT		▼	▼	▼	▼	▼	▲	▼	▼	▼	▼	▼	▼	▼
**Metabolism of proteins (R-MMU-392499)**	**7**	■		■	■		■		■	■		■		
**Signal Transduction (R-MMU-162582)**	**7**				■		■			■	■	■	■	■
Metabolism (R-MMU-1430728)	6	■		■		■	■		■	■				
Mitochondrial protein degradation (R-MMU-9837999)	4	■		■					■	■				
**Immune System (R-MMU-168256)**	**4**							■		■	■	■		
Citric acid cycle (TCA cycle) (R-MMU-71403)	3	■		■					■					
Pyruvate metabolism and Citric Acid (TCA) cycle (R-MMU-71406)	3	■		■					■					
Loss of proteins required for interphase microtubule ... (R-MMU-380284)	3									■			■	■
Loss of Nlp from mitotic centrosomes (R-MMU-380259)	3									■			■	■
AURKA Activation by TPX2 (R-MMU-8854518)	3									■			■	■
Centrosome maturation (R-MMU-380287)	3									■			■	■
Recruitment of mitotic centrosome proteins and complexes (R-MMU-380270)	3									■			■	■
Regulation of PLK1 Activity at G2/M Transition (R-MMU-2565942)	3									■			■	■
Recruitment of NuMA to mitotic centrosomes (R-MMU-380320)	3									■			■	■
Anchoring of the basal body to the plasma membrane (R-MMU-5620912)	3									■			■	■
The citric acid (TCA) cycle and respiratory electron transport	3	■		■					■					
G2/M Transition (R-MMU-69275)	3									■			■	■
Mitotic G2-G2/M phases (R-MMU-453274)	3									■			■	■
Cilium Assembly (R-MMU-5617833)	3									■			■	■
Mitotic Prometaphase (R-MMU-68877)	3									■			■	■
Organelle biogenesis and maintenance (R-MMU-1852241)	3									■			■	■
**M Phase (R-MMU-68886)**	**3**									■			■	■
**Signaling by Receptor Tyrosine Kinases (R-MMU-9006934)**	**3**									■	■	■		
**Cell Cycle, Mitotic (R-MMU-69278)**	**3**									■			■	■
Cell Cycle (R-MMU-1640170)	3									■			■	■
Transport of small molecules (R-MMU-382551)	3		■				■			■				

Continues on the next page.

Exclusively deregulated in PCPNIC *vs.* CT

REACTOME PATHWAYS<br>**FEMALES** (continuation)	# OF PTNS	Aco2	Atp1a2	Cs	Ctbp1	Gda	Gnb4	Hspa2	Idh3a	Prkaca	Sh3gl2	Rab4b	Ywhae	Ywhag
REGULATION: PCPNIC *vs.* CT		▼	▼	▼	▼	▼	▲	▼	▼	▼	▼	▼	▼	▼
Activation of BAD and translocation to mitochondria (R-MMU-111447)	2												■	■
Chk1/Chk2(Cds1) mediated inactivation of Cyclin B: ... (R-MMU-75035)	2												■	■
Activation of BH3-only proteins (R-MMU-114452)	2												■	■
RHO GTPases activate PKNs (R-MMU-5625740)	2												■	■
GPER1 signaling (R-MMU-9634597)	2						■			■				
Vasopressin regulates renal water homeostasis via ... (R-MMU-432040)	2						■			■				
Glucagon-like Peptide-1 (GLP1) regulates insulin secretion (R-MMU-381676)	2						■			■				
**Intrinsic Pathway for Apoptosis (R-MMU-109606)**	**2**						■			■				
Aquaporin-mediated transport (R-MMU-445717)	2						■			■				
**Ion homeostasis (R-MMU-5578775)**	**2**		■							■				
Regulation of insulin secretion (R-MMU-422356)	2						■			■				
**Regulation of HSF1-mediated heat shock response (R-MMU-3371453)**	**2**							■					■	
**Signaling by MET (R-MMU-6806834)**	**2**										■	■		
**Opioid Signalling (R-MMU-111885)**	**2**						■			■				
G2/M DNA damage checkpoint (R-MMU-69473)	2												■	■
TP53 Regulates Metabolic Genes (R-MMU-5628897)	2												■	■
Integration of energy metabolism (R-MMU-163685)	2						■			■				
**Cellular response to heat stress (R-MMU-3371556)**	**2**							■					■	
Cardiac conduction (R-MMU-5576891)	2		■							■				
**Apoptosis (R-MMU-109581)**	**2**												■	■
**Programmed Cell Death (R-MMU-5357801)**	**2**												■	■
G alpha (s) signalling events (R-MMU-418555)	2						■			■				
G2/M Checkpoints (R-MMU-69481)	2												■	■
Neurotransmitter receptors and postsynaptic signal ... (R-MMU-112314)	2						■			■				
**Muscle contraction (R-MMU-397014)**	**2**		■							■				
**Transmission across Chemical Synapses (R-MMU-112315)**	**2**						■			■				
Signaling by WNT (R-MMU-195721)	2				■		■							
**RHO GTPase Effectors (R-MMU-195258)**	**2**												■	■
Cell Cycle Checkpoints (R-MMU-69620)	2												■	■

Continues on the next page.

Exclusively deregulated in PCPNIC *vs.* CT

FEMALES (continuation)

REACTOME PATHWAYS	# OF PTNS	Aco2	Atp1a2	Cs	Ctbp1	Gda	Gnb4	Hspa2	Idh3a	Prkaca	Sh3gl2	Rab4b	Ywhae	Ywhag
REGULATION: PCPNIC *vs.* CT		▼	▼	▼	▼	▼	▲	▼	▼	▼	▼	▼	▼	▼
**Axon guidance (R-MMU-422475)**	**2**									■	■			
**Nervous system development (R-MMU-9675108)**	**2**									■	■			
G alpha (i) signalling events (R-MMU-418594)	2						■			■				
Transcriptional Regulation by TP53 (R-MMU-3700989)	2												■	■
**Neuronal System (R-MMU-112316)**	**2**						■			■				
CREB1 phosphorylation through the activation of ... (R-MMU-442720)	1									■				
PKA activation in glucagon signalling (R-MMU-164378)	1									■				
NADE modulates death signalling (R-MMU-205025)	1												■	
Glucagon signaling in metabolic regulation (R-MMU-163359)	1									■				
HDL assembly (R-MMU-8963896)	1									■				
HSF1 activation (R-MMU-3371511)	1												■	
Repression of WNT target genes (R-MMU-4641265)	1				■									
**MET receptor recycling (R-MMU-8875656)**	**1**											■		
Retrograde neurotrophin signalling (R-MMU-177504)	1										■			
Regulation of localization of FOXO transcription factors (R-MMU-9614399)	1													■
Attenuation phase (R-MMU-3371568)	1							■						
PKA activation (R-MMU-163615)	1									■				
Rap1 signalling (R-MMU-392517)	1									■				
PKA-mediated phosphorylation of CREB (R-MMU-111931)	1									■				
Post NMDA receptor activation events (R-MMU-438064)	1									■				
DARPP-32 events (R-MMU-180024)	1									■				
Purine catabolism (R-MMU-74259)	1					■								
G beta:gamma signalling through BTK (R-MMU-8964315)	1						■							
Plasma lipoprotein assembly (R-MMU-8963898)	1									■				
CD209 (DC-SIGN) signaling (R-MMU-5621575)	1									■				
Prostacyclin signalling through prostacyclin receptor (R-MMU-392851)	1						■							
Signaling by Hippo (R-MMU-2028269)	1												■	
G beta:gamma signalling through PLC beta (R-MMU-418217)	1						■							

Continues on the next page.

Exclusively deregulated in PCPNIC *vs.* CT

FEMALES (continuation)

REACTOME PATHWAYS	# OF PTNS	Aco2	Atp1a2	Cs	Ctbp1	Gda	Gnb4	Hspa2	Idh3a	Prkaca	Sh3gl2	Rab4b	Ywhae	Ywhag
**REGULATION: PCPNIC *vs.* CT**		▼	▼	▼	▼	▼	▲	▼	▼	▼	▼	▼	▼	▼
G beta:gamma signalling through CDC42 (R-MMU-8964616)	1						■							
Presynaptic function of Kainate receptors (R-MMU-500657)	1						■							
Negative regulation of MET activity (R-MMU-6807004)	1										■			
ADP signalling through P2Y purinoceptor 12 (R-MMU-392170)	1						■							
FOXO-mediated transcription (R-MMU-9614085)	1													■
Adrenaline,noradrenaline inhibits insulin secretion (R-MMU-400042)	1						■							
Thromboxane signalling through TP receptor (R-MMU-428930)	1						■							
G-protein activation (R-MMU-202040)	1						■							
**HSF1-dependent transactivation (R-MMU-3371571)**	**1**							■						
CaM pathway (R-MMU-111997)	1									■				
Calmodulin induced events (R-MMU-111933)	1									■				
ADP signalling through P2Y purinoceptor 1 (R-MMU-418592)	1						■							
G beta:gamma signalling through PI3Kgamma (R-MMU-392451)	1						■							
DAG and IP3 signaling (R-MMU-1489509)	1									■				
**Ca-dependent events (R-MMU-111996)**	**1**									■				
G protein gated Potassium channels (R-MMU-1296059)	1						■							
Activation of G protein gated Potassium channels (R-MMU-1296041)	1						■							
Inhibition of voltage gated Ca2+ channels via Gbeta ... (R-MMU-997272)	1						■							
Activation of kainate receptors upon glutamate binding (R-MMU-451326)	1						■							
EGFR downregulation (R-MMU-182971)	1										■			
**Thrombin signalling through proteinase activated ... (R-MMU-456926)**	**1**						■							
Deactivation of the beta-catenin transactivating complex (R-MMU-3769402)	1				■									
G-protein beta:gamma signalling (R-MMU-397795)	1						■							
Signal amplification (R-MMU-392518)	1						■							
Glucagon-type ligand receptors (R-MMU-420092)	1						■							
G alpha (z) signalling events (R-MMU-418597)	1						■							
Lysosome Vesicle Biogenesis (R-MMU-432720)	1										■			
Nucleotide catabolism (R-MMU-8956319)	1					■								
Inwardly rectifying K+ channels (R-MMU-1296065)	1						■							

Continues on the next page.

FIGURE 1e

Exclusively deregulated in PCPNIC *vs.* CT

FEMALES (continuation)

REACTOME PATHWAYS	# OF PTNS	Aco2	Atp1a2	Cs	Ctbp1	Gda	Gnb4	Hspa2	Idh3a	Prkaca	Sh3gl2	Rab4b	Ywhae	Ywhag
**REGULATION: PCPNIC *vs.* CT**		▼	▼	▼	▼	▼	▲	▼	▼	▼	▼	▼	▼	▼
**<u>PLC beta mediated events (R-MMU-112043)</u>**	**<u>1</u>**									■				
**<u>Recycling pathway of L1 (R-MMU-437239)</u>**	**<u>1</u>**										■			
**<u>Activation of NMDA receptors and postsynaptic events (R-MMU-442755)</u>**	**<u>1</u>**									■				
SUMOylation of transcription cofactors (R-MMU-3899300)	1				■									
Cooperation of PDCL (PhLP1) and TRiC/CCT in G-prot. ... (R-MMU-6814122)	1						■							
Protein folding (R-MMU-391251)	1						■							
Chaperonin-mediated protein folding (R-MMU-390466)	1						■							
RET signaling (R-MMU-8853659)	1									■				
**<u>G-protein mediated events (R-MMU-112040)</u>**	**<u>1</u>**									■				
GABA B receptor activation (R-MMU-977444)	1						■							
Activation of GABAB receptors (R-MMU-991365)	1						■							
**<u>Ca2+ pathway (R-MMU-4086398)</u>**	**<u>1</u>**						■							
Interleukin-3, Interleukin-5 and GM-CSF signaling (R-MMU-512988)	1									■				
Signaling by EGFR (R-MMU-177929)	1										■			
HSP90 chaperone cycle for steroid hormone receptors ... (R-MMU-3371497)	1							■						
**<u>Ion transport by P-type ATPases (R-MMU-936837)</u>**	**<u>1</u>**		■											
Golgi Associated Vesicle Biogenesis (R-MMU-432722)	1										■			
GLI3 is processed to GLI3R by the proteasome (R-MMU-5610785)	1									■				
Class B/2 (Secretin family receptors) (R-MMU-373080)	1						■							
GABA receptor activation (R-MMU-977443)	1						■							
**<u>RAB geranylgeranylation (R-MMU-8873719)</u>**	**<u>1</u>**											■		
**UNCLASSIFIED:**	5	Sntb1 (▲), Hdhd2 (▼), Ppp1r7 (▼), Ndrg2 (▼), Tppp (▼)												

Exclusively deregulated in PCPNIC *vs.* CT

MALES

REACTOME PATHWAYS	# OF PTNS	Acta1	Amph	Arf4	Arpc1a	Camk2a	Cbr1	Col6a6	Eif4a2	Mapk1	Mapk3	Rab4b	Stx1a	Uchl1
REGULATION: PCPNIC *vs.* CT		▲	▼	▼	▼	▲	▼	▼	▼	▲	▲	▲	▼	▲
**Immune System (R-MMU-168256)**	**7**				■	■			■	■	■	■	■	
**Signal Transduction (R-MMU-162582)**	**6**				■	■		■		■	■	■		
Cytokine Signaling in Immune system (R-MMU-1280215)	5					■			■	■	■		■	
Developmental Biology (R-MMU-1266738)	5				■			■		■	■		■	
Interferon Signaling (R-MMU-913531)	4					■			■	■	■			
**Axon guidance (R-MMU-422475)**	**4**				■			■		■	■			
**Nervous system development (R-MMU-9675108)**	**4**				■			■		■	■			
**Signaling by Receptor Tyrosine Kinases (R-MMU-9006934)**	**4**							■		■	■	■		
Innate Immune System (R-MMU-168249)	4				■					■	■	■		
**Metabolism of proteins (R-MMU-392499)**	**4**			■					■			■		■
Interferon gamma signaling (R-MMU-877300)	3					■				■	■			
RHO GTPases Activate WASPs and WAVEs (R-MMU-5663213)	3				■					■	■			
NCAM signaling for neurite out-growth (R-MMU-375165)	3							■		■	■			
**Cellular response to heat stress (R-MMU-3371556)**	**3**					■				■	■			
Regulation of actin dynamics for phagocytic cup formation (R-MMU-2029482)	3				■					■	■			
Fcgamma receptor (FCGR) dependent phagocytosis (R-MMU-2029480)	3				■					■	■			
**RHO GTPase Effectors (R-MMU-195258)**	**3**				■					■	■			
RAF/MAP kinase cascade (R-MMU-5673001)	3					■				■	■			
MAPK1/MAPK3 signaling (R-MMU-5684996)	3					■				■	■			
Signaling by Interleukins (R-MMU-449147)	3									■	■		■	
MAPK family signaling cascades (R-MMU-5683057)	3					■				■	■			
Cellular responses to stress (R-MMU-2262752)	3					■				■	■			
Cellular responses to stimuli (R-MMU-8953897)	3					■				■	■			
Membrane Trafficking (R-MMU-199991)	3		■	■	■									
Signaling by Rho GTPases (R-MMU-194315)	3				■					■	■			
Signaling by Rho GTPases, Miro GTPases and RHOBTB3 (R-MMU-9716542)	3				■					■	■			
Vesicle-mediated transport (R-MMU-5653656)	3		■	■	■									
Signaling by NODAL (R-MMU-1181150)	2									■	■			
Negative feedback regulation of MAPK pathway (R-MMU-5674499)	2									■	■			

Continues on the next page.

Exclusively deregulated in PCPNIC *vs.* CT

REACTOME PATHWAYS	# OF PTNS	GENES												
**MALES** (continuation)		Acta1	Amph	Arf4	Arpc1a	Camk2a	Cbr1	Col6a6	Eif4a2	Mapk1	Mapk3	Rab4b	Stx1a	Uchl1
**REGULATION: PCPNIC *vs.* CT**		▲	▼	▼	▼	▲	▼	▼	▼	▲	▲	▲	▼	▲
IFNG signaling activates MAPKs (R-MMU-9732724)	2									■	■			
Regulation of the apoptosome activity (R-MMU-9627069)	2									■	■			
Formation of apoptosome (R-MMU-111458)	2									■	■			
Activation of the AP-1 family of transcription factors (R-MMU-450341)	2									■	■			
Signal attenuation (R-MMU-74749)	2									■	■			
Golgi Cisternae Pericentriolar Stack Reorganization (R-MMU-162658)	2									■	■			
Frs2-mediated activation (R-MMU-170968)	2									■	■			
Cytochrome c-mediated apoptotic response (R-MMU-111461)	2									■	■			
Growth hormone receptor signaling (R-MMU-982772)	2									■	■			
ERKs are inactivated (R-MMU-202670)	2									■	■			
Prolonged ERK activation events (R-MMU-169893)	2									■	■			
Signaling by Activin (R-MMU-1502540)	2									■	■			
Apoptotic factor-mediated response (R-MMU-111471)	2									■	■			
Spry regulation of FGF signaling (R-MMU-1295596)	2									■	■			
Oncogene Induced Senescence (R-MMU-2559585)	2									■	■			
Gastrin-CREB signalling pathway via PKC and MAPK (R-MMU-881907)	2									■	■			
ERK/MAPK targets (R-MMU-198753)	2									■	■			
Signal transduction by L1 (R-MMU-445144)	2									■	■			
RAF-independent MAPK1/3 activation (R-MMU-112409)	2									■	■			
RHO GTPases Activate NADPH Oxidases (R-MMU-5668599)	2									■	■			
Signalling to ERKs (R-MMU-187687)	2									■	■			
SMAD2/SMAD3:SMAD4 heterotrimer regulates transcription	2									■	■			
Downregulation of SMAD2/3:SMAD4 transcriptional activity (R-MMU-2173795)	2									■	■			
Nuclear Events (kinase and transcription factor activation) (R-MMU-198725)	2									■	■			
MAPK targets/ Nuclear events mediated by MAP kinases (R-MMU-450282)	2									■	■			
Negative regulation of FGFR3 signaling (R-MMU-5654732)	2									■	■			
ISG15 antiviral mechanism (R-MMU-1169408)	2								■		■			
Negative regulation of FGFR4 signaling (R-MMU-5654733)	2									■	■			
**Thrombin signalling through proteinase activated ... (R-MMU-456926)**	**2**									■	■			

Continues on the next page.

FIGURE 1h

Exclusively deregulated in PCPNIC *vs.* CT

REACTOME PATHWAYS<br>**MALES** (continuation)	# OF PTNS	GENES<br>Acta1	Amph	Arf4	Arpc1a	Camk2a	Cbr1	Col6a6	Eif4a2	Mapk1	Mapk3	Rab4b	Stx1a	Uchl1
**REGULATION: PCPNIC *vs.* CT**		▲	▼	▼	▼	▲	▼	▼	▼	▲	▲	▲	▼	▲
Negative regulation of FGFR1 signaling (R-MMU-5654726)	2									■	■			
Negative regulation of FGFR2 signaling (R-MMU-5654727)	2									■	■			
Oxidative Stress Induced Senescence (R-MMU-2559580)	2									■	■			
**Intrinsic Pathway for Apoptosis (R-MMU-109606)**	**2**									■	■			
Senescence-Associated Secretory Phenotype (SASP) (R-MMU-2559582)	2									■	■			
Signaling by FGFR3 (R-MMU-5654741)	2									■	■			
MAP2K and MAPK activation (R-MMU-5674135)	2									■	■			
Signaling by FGFR4 (R-MMU-5654743)	2									■	■			
Transcriptional activity of SMAD2/SMAD3:SMAD4 ... (R-MMU-2173793)	2									■	■			
Negative regulation of MAPK pathway (R-MMU-5675221)	2									■	■			
Insulin receptor signalling cascade (R-MMU-74751)	2									■	■			
Signaling by FGFR1 (R-MMU-5654736)	2									■	■			
MAP kinase activation (R-MMU-450294)	2									■	■			
Interleukin-17 signaling (R-MMU-448424)	2									■	■			
**Regulation of HSF1-mediated heat shock response (R-MMU-3371453)**	**2**									■	■			
Signaling by NTRK1 (TRKA) (R-MMU-187037)	2									■	■			
Signaling by FGFR2 (R-MMU-5654738)	2									■	■			
L1CAM interactions (R-MMU-373760)	2									■	■			
Signaling by NTRKs (R-MMU-166520)	2									■	■			
Signaling by Insulin receptor (R-MMU-74752)	2									■	■			
Signaling by TGF-beta Receptor Complex (R-MMU-170834)	2									■	■			
Toll Like Receptor 3 (TLR3) Cascade (R-MMU-168164)	2									■	■			
Signaling by FGFR (R-MMU-190236)	2									■	■			
MyD88 cascade initiated on plasma membrane (R-MMU-975871)	2									■	■			
Toll Like Receptor 5 (TLR5) Cascad (R-MMU-168176)	2									■	■			
Toll Like Receptor 10 (TLR10) Cascade (R-MMU-168142)	2									■	■			
Toll Like Receptor 2 (TLR2) Cascade (R-MMU-181438)	2									■	■			
MyD88:MAL(TIRAP) cascade initiated on plasma membrane	2									■	■			
Toll Like Receptor TLR6:TLR2 Cascade (R-MMU-168188)	2									■	■			

Continues on the next page.

Exclusively deregulated in PCPNIC *vs.* CT

MALES (continuation)

REACTOME PATHWAYS	# OF PTNS	Acta1	Amph	Arf4	Arpc1a	Camk2a	Cbr1	Col6a6	Eif4a2	Mapk1	Mapk3	Rab4b	Stx1a	Uchl1
**REGULATION: PCPNIC *vs.* CT**		▲	▼	▼	▼	▲	▼	▼	▼	▲	▲	▲	▼	▲
Toll Like Receptor TLR1:TLR2 Cascade (R-MMU-168179)	2									■	■			
TRAF6 mediated induction of NFkB and MAP kinases ... (R-MMU-975138)	2									■	■			
MyD88 dependent cascade initiated on endosome (R-MMU-975155)	2									■	■			
Toll Like Receptor 7/8 (TLR7/8) Cascade (R-MMU-168181)	2									■	■			
Antiviral mechanism by IFN-stimulated genes (R-MMU-1169410)	2								■		■			
Toll Like Receptor 9 (TLR9) Cascade (R-MMU-168138)	2									■	■			
FCERI mediated MAPK activation (R-MMU-2871796)	2									■	■			
MyD88-independent TLR4 cascade (R-MMU-166166)	2									■	■			
TRIF(TICAM1)-mediated TLR4 signaling (R-MMU-937061)	2									■	■			
Mitotic Prophase (R-MMU-68875)	2									■	■			
**Apoptosis (R-MMU-109581)**	**2**									■	■			
PI5P, PP2A and IER3 Regulate PI3K/AKT Signaling (R-MMU-6811558)	2									■	■			
Signaling by TGFB family members (R-MMU-9006936)	2									■	■			
Negative regulation of the PI3K/AKT network (R-MMU-199418)	2									■	■			
Toll Like Receptor 4 (TLR4) Cascade (R-MMU-166016)	2									■	■			
Cellular Senescence (R-MMU-2559583)	2									■	■			
**Programmed Cell Death (R-MMU-5357801)**	**2**									■	■			
Clathrin-mediated endocytosis (R-MMU-8856828)	2		■		■									
Toll-like Receptor Cascades (R-MMU-168898)	2									■	■			
**Muscle contraction (R-MMU-397014)**	**2**	■				■								
Fc epsilon receptor (FCERI) signaling (R-MMU-2454202)	2									■	■			
G alpha (q) signalling events (R-MMU-416476)	2									■	■			
**Transmission across Chemical Synapses (R-MMU-112315)**	**2**					■							■	
PIP3 activates AKT signaling (R-MMU-1257604)	2									■	■			
Intracellular signaling by second messengers (R-MMU-9006925)	2									■	■			
Platelet activation, signaling and aggregation (R-MMU-76002)	2									■	■			
**Neuronal System (R-MMU-112316)**	**2**					■							■	
**M Phase (R-MMU-68886)**	**2**									■	■			
**Cell Cycle, Mitotic (R-MMU-69278)**	**2**									■	■			

Continues on the next page.

Exclusively deregulated in PCPNIC *vs.* CT

MALES (continuation)

REACTOME PATHWAYS	# OF PTNS	Acta1	Amph	Arf4	Arpc1a	Camk2a	Cbr1	Col6a6	Eif4a2	Mapk1	Mapk3	Rab4b	Stx1a	Uchl1
**REGULATION: PCPNIC *vs.* CT**		▲	▼	▼	▼	▲	▼	▼	▼	▲	▲	▲	▼	▲
Phospho-PLA2 pathway (R-MMU-111995)	1									■				
RNA Polymerase I Promoter Opening (R-MMU-73728)	1										■			
Estrogen-stimulated signaling through PRKCZ (R-MMU-9634635)	1									■				
MAPK1 (ERK2) activation (R-MMU-112411)	1									■				
Estrogen-dependent nuclear events downstream of ... (R-MMU-9634638)	1									■				
MAPK3 (ERK1) activation (R-MMU-110056)	1										■			
**<u>MET receptor recycling (R-MMU-8875656)</u>**	**<u>1</u>**											■		
LGI-ADAM interactions (R-MMU-5682910)	1												■	
Synthesis of Prostaglandins (PG) and Thromboxanes (TX) (R-MMU-2162123)	1						■							
Other interleukin signaling (R-MMU-449836)	1												■	
Insertion of tail-anchored proteins into the endoplasmic ... (R-MMU-9609523)	1												■	
Acetylcholine Neurotransmitter Release Cycle (R-MMU-264642)	1												■	
Norepinephrine Neurotransmitter Release Cycle (R-MMU-181430)	1												■	
Serotonin Neurotransmitter Release Cycle (R-MMU-181429)	1												■	
GABA synthesis, release, reuptake and degradation (R-MMU-888590)	1												■	
NCAM1 interactions (R-MMU-419037)	1							■						
VxPx cargo-targeting to cilium (R-MMU-5620916)	1			■										
Unblocking of NMDA receptors, glutamate binding and ... (R-MMU-438066)	1					■								
Deadenylation of mRNA (R-MMU-429947)	1								■					
Dopamine Neurotransmitter Release Cycle (R-MMU-212676)	1												■	
Glutamate Neurotransmitter Release Cycle (R-MMU-210500)	1												■	
**<u>HSF1-dependent transactivation (R-MMU-3371571)</u>**	**<u>1</u>**					■								
**<u>Ca-dependent events (R-MMU-111996)</u>**	**<u>1</u>**									■				
Glutamate binding, activation of AMPA receptors and ... (R-MMU-399721)	1					■								
Trafficking of AMPA receptors (R-MMU-399719)	1					■								
RAF activation (R-MMU-5673000)	1					■								
Striated Muscle Contraction (R-MMU-390522)	1	■												
**<u>PLC beta mediated events (R-MMU-112043)</u>**	**<u>1</u>**									■				
**<u>Recycling pathway of L1 (R-MMU-437239)</u>**	**<u>1</u>**									■				

Continues on the next page.

Exclusively deregulated in PCPNIC *vs.* CT

REACTOME PATHWAYS	# OF PTNS	GENES: Acta1	Amph	Arf4	Arpc1a	Camk2a	Cbr1	Col6a6	Eif4a2	Mapk1	Mapk3	Rab4b	Stx1a	Uchl1
**MALES** (continuation)														
REGULATION: PCPNIC *vs.* CT		▲	▼	▼	▼	▲	▼	▼	▼	▲	▲	▲	▼	▲
**Activation of NMDA receptors and postsynaptic events (R-MMU-442755)**	**1**					■								
EPHB-mediated forward signaling (R-MMU-3928662)	1				■									
Cargo trafficking to the periciliary membrane (R-MMU-5620920)	1			■										
**G-protein mediated events (R-MMU-112040)**	**1**									■				
Collagen chain trimerization (R-MMU-8948216)	1							■						
**Ca2+ pathway (R-MMU-4086398)**	**1**					■								
RNA Polymerase I Promoter Clearance (R-MMU-73854)	1										■			
RNA Polymerase I Transcription (R-MMU-73864)	1										■			
ECM proteoglycans (R-MMU-3000178)	1							■						
Assembly of collagen fibrils and other multimeric structures (R-MMU-2022090)	1							■						
**Ion homeostasis (R-MMU-5578775)**	**1**					■								
Neurotransmitter release cycle (R-MMU-112310)	1												■	
Collagen degradation (R-MMU-1442490)	1							■						
Deadenylation-dependent mRNA decay (R-MMU-429914)	1								■					
**Ion transport by P-type ATPases (R-MMU-936837)**	**1**					■								
Ribosomal scanning and start codon recognition (R-MMU-72702)	1								■					
Signaling by PDGF (R-MMU-186797)	1							■						
Translation initiation complex formation (R-MMU-72649)	1								■					
Activation of the mRNA upon binding of the cap-binding ... (R-MMU-72662)	1								■					
Arachidonic acid metabolism (R-MMU-2142753)	1						■							
Collagen biosynthesis and modifying enzymes (R-MMU-1650814)	1							■						
**RAB geranylgeranylation (R-MMU-8873719)**	**1**											■		
**Signaling by MET (R-MMU-6806834)**	**1**											■		
EPH-Ephrin signaling (R-MMU-2682334)	1				■									
Extra-nuclear estrogen signaling (R-MMU-9009391)	1									■				
**Opioid Signalling (R-MMU-111885)**	**1**									■				
**UNCLASSIFIED:**	2	Cfap20 (▼), Phf24 (▼)												

Commonly deregulated in NIC *vs.* CT and PCPNIC *vs.* CT

Add2: Beta-adducin; **Aldoa**: Fructose-bisphosphate aldolase A; **Basp1**: Brain acid soluble protein 1; **Camk2g**: Calcium/calmodulin-dependent protein kinase type II subunit gamma; **Camkv**: CaM kinase-like vesicle-associated protein; **Cct5**: T-complex protein 1 subunit epsilon; **Dbn1**: Drebrin; **Gad2**: Glutamate decarboxylase 2; **Gstp1**: Glutathione S-transferase P 1; **Hnrnpk**: Heterogeneous nuclear ribonucleoprotein K; **Ldhb**: L-lactate dehydrogenase B chain; **Mapt**: Microtubule-associated protein tau; **Pkm**: Pyruvate kinase PKM; **Prrt2**: Proline-rich transmembrane protein 2; **Psap**: Prosaposin; **Rab8a**: Ras-related protein Rab-8A; **Septin7**: Septin-7; **Ywhab**: 14-3-3 protein beta/alph.

REACTOME PATHWAYS	# OF PTNS	GENES											
**FEMALES**		Aldoa	Basp1	Camk2g	Cct5	Gad2	Gstp1	Ldhb	Mapt	Pkm	Rab8a	Septin7	Ywhab
**REGULATION:** **NIC *vs.* CT**		▼	▼	▲	▼	▼	▼	▼	▼	▼	▼	▼	▼
**PCPNIC *vs.* CT**		▼	▼	▲	▼	▼	▼	▼	▼	▼	▲	▼	▼
Immune System (R-MMU-168256)	6	■		■			■		■	■			■
Metabolism (R-MMU-1430728)	4	■					■	■		■			
MAPK family signaling cascades (R-MMU-5683057)	3			■								■	■
Neutrophil degranulation (R-MMU-6798695)	3	■					■			■			
Innate Immune System (R-MMU-168249)	3	■					■			■			
RAF activation (R-MMU-5673000)	2			■									■
Glycolysis (R-MMU-70171)	2	■								■			
Glucose metabolism (R-MMU-70326)	2	■								■			
Apoptosis (R-MMU-109581)	2								■				■
Programmed Cell Death (R-MMU-5357801)	2								■				■
Interferon Signaling (R-MMU-913531)	2			■					■				
Transmission across Chemical Synapses (R-MMU-112315)	2			■		■							
Metabolism of carbohydrates (R-MMU-71387)	2	■								■			
RAF/MAP kinase cascade (R-MMU-5673001)	2			■									■
MAPK1/MAPK3 signaling (R-MMU-5684996)	2			■									■
Neuronal System (R-MMU-112316)	2			■		■							
Cytokine Signaling in Immune system (R-MMU-1280215)	2			■					■				
Cellular responses to stress (R-MMU-2262752)	2			■			■						
Cellular responses to stimuli (R-MMU-8953897)	2			■			■						
GABA synthesis (R-MMU-888568)	1					■							
Association of TriC/CCT with target proteins during ... (R-MMU-390471)	1				■								
Frs2-mediated activation (R-MMU-170968)	1												■
Regulation of localization of FOXO transcription factors (R-MMU-9614399)	1												■
Activation of BAD and translocation to mitochondria (R-MMU-111447)	1												■
Chk1/Chk2(Cds1) mediated inactivation of Cyclin B: ... (R-MMU-75035)	1												■
Caspase-mediated cleavage of cytoskeletal proteins (R-MMU-264870)	1								■				
Prolonged ERK activation events (R-MMU-169893)	1												■

Continues on the next page.

Commonly deregulated in NIC *vs.* CT and PCPNIC *vs.* CT

FEMALES (continuation)

REACTOME PATHWAYS	# OF PTNS	Aldoa	Basp1	Camk2g	Cct5	Gad2	Gstp1	Ldhb	Mapt	Pkm	Rab8a	Septin7	Ywhab
**REGULATION: NIC *vs.* CT**		▼	▼	▲	▼	▼	▼	▼	▼	▼	▼	▼	▼
**PCPNIC *vs.* CT**		▼	▼	▲	▼	▼	▼	▼	▼	▼	▲	▼	▼
Rap1 signalling (R-MMU-392517)	1												■
Butyrate Response Factor 1 (BRF1) binds and ... (R-MMU-450385)	1												■
Tristetraprolin (TTP, ZFP36) binds and destabilizes mRNA (R-MMU-450513)	1												■
Activation of BH3-only proteins (R-MMU-114452)	1												■
GABA synthesis, release, reuptake and degradation (R-MMU-888590)	1					■							
Signaling by Hippo (R-MMU-2028269)	1												■
Interferon gamma signaling (R-MMU-877300)	1			■									
VxPx cargo-targeting to cilium (R-MMU-5620916)	1										■		
Unblocking of NMDA receptors, glutamate binding and ... (R-MMU-438066)	1												
RHO GTPases activate PKNs (R-MMU-5625740)	1												■
FOXO-mediated transcription (R-MMU-9614085)	1												■
HSF1-dependent transactivation (R-MMU-3371571)	1			■									
mTORC1-mediated signalling (R-MMU-166208)	1												■
Signalling to ERKs (R-MMU-187687)	1												■
Pyruvate metabolism (R-MMU-70268)	1							■					
Glutamate binding, activation of AMPA receptors and ... (R-MMU-399721)	1			■									
Trafficking of AMPA receptors (R-MMU-399719)	1			■									
Gluconeogenesis (R-MMU-70263)	1	■											
TBC/RABGAPs (R-MMU-8854214)	1										■		
Detoxification of Reactive Oxygen Species (R-MMU-3299685)	1						■						
Activation of NMDA receptors and postsynaptic events (R-MMU-442755)	1			■									
Paracetamol ADME (R-MMU-9753281)	1						■						
Apoptotic cleavage of cellular proteins (R-MMU-111465)	1								■				
Glutathione conjugation (R-MMU-156590)	1						■						
Cooperation of PDCL (PhLP1) and TRiC/CCT in G-prot. ... (R-MMU-6814122)	1				■								
Intrinsic Pathway for Apoptosis (R-MMU-109606)	1												■
Protein folding (R-MMU-391251)	1				■								
Chaperonin-mediated protein folding (R-MMU-390466)	1				■								

Continues on the next page.

FIGURE 2c

Commonly deregulated in NIC *vs.* CT and PCPNIC *vs.* CT

REACTOME PATHWAYS<br>**FEMALES** (continuation)	# OF PTNS	Aldoa	Basp1	Camk2g	Cct5	Gad2	Gstp1	Ldhb	Mapt	Pkm	Rab8a	Septin7	Ywhab
**REGULATION: NIC *vs.* CT**		▼	▼	▲	▼	▼	▼	▼	▼	▼	▼	▼	▼
**PCPNIC *vs.* CT**		▼	▼	▲	▼	▼	▼	▼	▼	▼	▲	▼	▼
Cargo trafficking to the periciliary membrane (R-MMU-5620920)	1										■		
MAP2K and MAPK activation (R-MMU-5674135)	1												■
RHOF GTPase cycle (R-MMU-9035034)	1		■										
MTOR signalling (R-MMU-165159)	1												■
Negative regulation of MAPK pathway (R-MMU-5675221)	1												■
Apoptotic execution phase (R-MMU-75153)	1								■				
Ion homeostasis (R-MMU-5578775)	1			■									
Neurotransmitter release cycle (R-MMU-112310)	1					■							
Pyruvate metabolism and Citric Acid (TCA) cycle (R-MMU-71406)	1							■					
Ion transport by P-type ATPases (R-MMU-936837)	1			■									
RAB geranylgeranylation (R-MMU-8873719)	1										■		
Signaling by NTRK1 (TRKA) (R-MMU-187037)	1												■
PKR-mediated signaling (R-MMU-9833482)	1								■				
G2/M DNA damage checkpoint (R-MMU-69473)	1												■
MAPK6/MAPK4 signaling (R-MMU-5687128)	1											■	
**UNCLASSIFIED:**	3	Prrt2 (▼,▼), Dbn1 (▼,▼), Camkv (▼,▼)											

Commonly deregulated in NIC *vs.* CT and PCPNIC *vs.* CT

MALES

REACTOME PATHWAYS	# OF PTNS	GENES: Add2	Psap	Hnrnpk
**REGULATION:** **NIC *vs.* CT**		▼	▲	▼
**PCPNIC *vs.* CT**		▼	▲	▼
Miscellaneous transport and binding events (R-MMU-5223345)	1	■		
Glycosphingolipid catabolism (R-MMU-9840310)	1		■	
SUMOylation of RNA binding proteins (R-MMU-4570464)	1			■
Glycosphingolipid metabolism (R-MMU-1660662)	1		■	
Sphingolipid metabolism (R-MMU-428157)	1		■	
Platelet degranulation (R-MMU-114608)	1		■	
Response to elevated platelet cytosolic Ca2+ (R-MMU-76005)	1		■	
SUMO E3 ligases SUMOylate target proteins (R-MMU-3108232)	1			■
SUMOylation (R-MMU-2990846)	1			■
Peptide ligand-binding receptors (R-MMU-375276)	1		■	
mRNA Splicing - Major Pathway (R-MMU-72163)	1			■
mRNA Splicing (R-MMU-72172)	1			■
Platelet activation, signaling and aggregation (R-MMU-76002)	1		■	
Processing of Capped Intron-Containing Pre-mRNA (R-MMU-72203)	1			■
G alpha (i) signalling events (R-MMU-418594)	1		■	
Class A/1 (Rhodopsin-like receptors) (R-MMU-373076)	1		■	

Commonly deregulated in PCP *vs.* CT and PCPNIC *vs.* CT

Actc1: Actin_alpha cardiac muscle 1; **Aldoc**: Fructose-bisphosphate aldolase C. ; **Arpc4**: Actin-related protein 2/3 complex subunit 4; **Atp6v1b1**: V-type proton ATPase subunit B_ kidney isoform; **Cfap61**: Cilia- and flagella-associated protein 61; **Copg2**: Coatomer subunit gamma-2; **Eef1g**: Elongation factor 1-gamma; **Eif4a2**: Eukaryotic initiation factor 4A-II; **Gnas**: Guanine nucleotide-binding protein G(s) subunit alpha isoforms XLas; **Ncam1**: Neural cell adhesion molecule 1; **Psap**: Prosaposin; **Pura**: Transcriptional activator protein Pur-alpha; **Rab10**: Ras-related protein Rab-10; **Rab3d**: Ras-related protein Rab-3D; **Rab8b**: Ras-related protein Rab-8B; **Rap1gds1**: Isoform 3 of Rap1 GTPase-GDP dissociation stimulator 1; **Syt1**: Synaptotagmin-1.

FEMALES

REACTOME PATHWAYS	# OF PTNS	GENES: Actc1	Aldoc	Arpc4	Atp6v1b1	Copg2	Eef1g	Eif4a2	Gnas	Ncam1	Psap	Rab10	Rab3d	Rab8b	Syt1
**REGULATION:** **PCP *vs.* CT**		▼	▼	▼	▼	▲	▼	▼	▼	▼	▼	▲	▼	▲	▲
**PCPNIC *vs.* CT**		▼	▼	▼	▼	▲	▼	▼	▼	▼	▼	▲	▼	▲	▼
Immune System (R-MMU-168256)	7		■	■	■			■			■	■	■		
Innate Immune System (R-MMU-168249)	6		■	■	■						■	■	■		
Metabolism of proteins (R-MMU-392499)	6					■	■	■				■	■	■	
Signal Transduction (R-MMU-162582)	6	■		■	■				■	■	■				
Membrane Trafficking (R-MMU-199991)	5			■		■						■		■	■
Vesicle-mediated transport (R-MMU-5653656)	5			■		■						■		■	■
Neutrophil degranulation (R-MMU-6798695)	4		■								■	■	■		
Post-translational protein modification (R-MMU-597592)	4					■						■	■	■	
RAB geranylgeranylation (R-MMU-8873719)	3											■	■	■	
**Nervous system development (R-MMU-9675108)**	**2**			■						■					
RAB GEFs exchange GTP for GDP on RABs (R-MMU-8876198)	2											■		■	
Rab regulation of trafficking (R-MMU-9007101)	2											■		■	
Clathrin-mediated endocytosis (R-MMU-8856828)	2			■											■
Translation (R-MMU-72766)	2						■	■							
**Axon guidance (R-MMU-422475)**	**2**			■						■					
GPCR ligand binding (R-MMU-500792)	2								■		■				
Eukaryotic Translation Elongation (R-MMU-156842)	1						■								
Acetylcholine Neurotransmitter Release Cycle (R-MMU-264642)	1														■
Norepinephrine Neurotransmitter Release Cycle (R-MMU-181430)	1														■
Serotonin Neurotransmitter Release Cycle (R-MMU-181429)	1														■
GABA synthesis, release, reuptake and degradation (R-MMU-888590)	1														■
Prostacyclin signalling through prostacyclin receptor (R-MMU-392851)	1								■						
**NCAM1 interactions (R-MMU-419037)**	**1**									■					
**Signal transduction by L1 (R-MMU-445144)**	**1**									■					
Deadenylation of mRNA (R-MMU-429947)	1							■							
Dopamine Neurotransmitter Release Cycle (R-MMU-212676)	1														■
Glutamate Neurotransmitter Release Cycle (R-MMU-210500)	1														■

Continues on the next page.

FIGURE 3b

Commonly deregulated in PCP *vs.* CT and PCPNIC *vs.* CT

REACTOME PATHWAYS<br>**FEMALES** (continuation)	# OF PTNS	Actc1	Aldoc	Arpc4	Atp6v1b1	Copg2	Eef1g	Eif4a2	Gnas	Ncam1	Psap	Rab10	Rab3d	Rab8b	Syt1
**REGULATION: PCP *vs.* CT**		▼	▼	▼	▼	▲	▼	▼	▼	▼	▼	▲	▼	▲	▲
**PCPNIC *vs.* CT**		▼	▼	▼	▼	▲	▼	▼	▼	▼	▼	▲	▼	▲	▼
GPER1 signaling (R-MMU-9634597)	1								■						
Insulin receptor recycling (R-MMU-77387)	1				■										
ISG15 antiviral mechanism (R-MMU-1169408)	1							■							
Transferrin endocytosis and recycling (R-MMU-917977)	1				■										
Vasopressin regulates renal water homeostasis via ... (R-MMU-432040)	1								■						
Glucagon-like Peptide-1 (GLP1) regulates insulin secretion (R-MMU-381676)	1								■						
Glucagon-type ligand receptors (R-MMU-420092)	1								■						
Gluconeogenesis (R-MMU-70263)	1		■												
Glycosphingolipid catabolism (R-MMU-9840310)	1										■				
RHO GTPases Activate WASPs and WAVEs (R-MMU-5663213)	1			■											
TBC/RABGAPs (R-MMU-8854214)	1													■	
Striated Muscle Contraction (R-MMU-390522)	1	■													
ROS and RNS production in phagocytes (R-MMU-1222556)	1				■										
EPHB-mediated forward signaling (R-MMU-3928662)	1			■											
Aquaporin-mediated transport (R-MMU-445717)	1								■						
**NCAM signaling for neurite out-growth (R-MMU-375165)**	**1**									■					
Neurotransmitter release cycle (R-MMU-112310)	1														■
Glycosphingolipid metabolism (R-MMU-1660662)	1										■				
Deadenylation-dependent mRNA decay (R-MMU-429914)	1							■							
Amino acids regulate mTORC1 (R-MMU-9639288)	1				■										
Cellular response to starvation (R-MMU-9711097)	1				■										
Iron uptake and transport (R-MMU-917937)	1				■										
Ribosomal scanning and start codon recognition (R-MMU-72702)	1							■							
Translation initiation complex formation (R-MMU-72649)	1							■							
Class B/2 (Secretin family receptors) (R-MMU-373080)	1								■						
Activation of the mRNA upon binding of the cap-binding ... (R-MMU-72662)	1							■							
Glycolysis (R-MMU-70171)	1		■												
Regulation of insulin secretion (R-MMU-422356)	1								■						
**UNCLASSIFIED:**	3	Cfap61 (▼,▼), Pura (▼,▼), Rap1gds1 (▼,▼)													

Commonly deregulated in PCP *vs.* CT and PCPNIC *vs.* CT

REACTOME PATHWAYS	# OF PTNS	GENE
**MALES**		Ncam1
**REGULATION:** **PCP *vs.* CT**		▼
**PCPNIC *vs.* CT**		▼
**NCAM1 interactions (R-MMU-419037)**	**1**	
**Signal transduction by L1 (R-MMU-445144)**	**1**	
**NCAM signaling for neurite out-growth (R-MMU-375165)**	**1**	
L1CAM interactions (R-MMU-373760)	1	
**Axon guidance (R-MMU-422475)**	**1**	
**Nervous system development (R-MMU-9675108)**	**1**	
RAF/MAP kinase cascade (R-MMU-5673001)	1	
MAPK1/MAPK3 signaling (R-MMU-5684996)	1	
MAPK family signaling cascades (R-MMU-5683057)	1	
Developmental Biology (R-MMU-1266738)	1	

Commonly deregulated in all groups *vs.* CT

Actr3b: Actin-related protein 3B; **Add2**: Beta-adducin; **C1qbp**: Complement component 1 Q subcomponent-binding protein_ mitochondrial; **Calb2**: Calretinin; **Cbr1**: Carbonyl reductase [NADPH] 1; **Ckmt2**: Creatine kinase S-type_ mitochondrial; **Hspd1**: 60 kDa heat shock protein_ mitochondrial; **Naxe**: NAD(P)H-hydrate epimerase; **Sncb**: Beta-synuclein; **Stat3**: Signal transducer and activator of transcription 3.

FEMALES

REACTOME PATHWAYS	# OF PTNS	Add2	C1qbp	Cbr1	Ckmt2	Hspd1	Naxe	Stat3
**REGULATION: NIC *vs.* CT**		▲	▲	▼	▲	▼	▲	▼
**PCP *vs.* CT**		▼	▲	▼	▲	▼	▲	▼
**PCPNIC *vs.* CT**		▼	▲	▼	▲	▼	▲	▼
Metabolism (R-MMU-1430728)	3			■	■		■	
Interleukin-37 signaling (R-MMU-9008059)	1							■
MET activates STAT3 (R-MMU-8875791)	1							■
PTK6 Activates STAT3 (R-MMU-8849474)	1							■
STAT3 nuclear events downstream of ALK signaling (R-MMU-9701898)	1							■
Interleukin-10 signaling (R-MMU-6783783)	1							■
Interleukin-23 signaling (R-MMU-9020933)	1							■
Interleukin-9 signaling (R-MMU-8985947)	1							■
Mitochondrial protein import (R-MMU-1268020)	1					■		
Interleukin-21 signaling (R-MMU-9020958)	1							■
Creatine metabolism (R-MMU-71288)	1				■			
Interleukin-6 signaling (R-MMU-1059683)	1							■
Interleukin-27 signaling (R-MMU-9020956)	1							■
Interleukin-35 Signalling (R-MMU-8984722)	1							■
Interleukin-15 signaling (R-MMU-8983432)	1							■
Synthesis of Prostaglandins (PG) and Thromboxanes (TX) (R-MMU-2162123)	1			■				
Interleukin-4 and Interleukin-13 signaling (R-MMU-6785807)	1							■
Interleukin-7 signaling (R-MMU-1266695)	1							■
Interleukin-12 family signaling (R-MMU-447115)	1							■
Nicotinamide salvaging (R-MMU-197264)	1						■	
Signaling by ALK (R-MMU-201556)	1							■
Interleukin-20 family signaling (R-MMU-8854691)	1							■
Intrinsic Pathway of Fibrin Clot Formation (R-MMU-140837)	1		■					
Interleukin-6 family signaling (R-MMU-6783589)	1							■
Miscellaneous transport and binding events (R-MMU-5223345)	1	■						
Downstream signal transduction (R-MMU-186763)	1							■

Continues on the next page.

Commonly deregulated in all groups *vs.* CT

REACTOME PATHWAYS<br>**FEMALES** (continuation)	# OF PTNS	Add2	C1qbp	Cbr1	Ckmt2	Hspd1	Naxe	Stat3
**REGULATION: NIC *vs.* CT**		▲	▲	▼	▲	▼	▲	▼
**PCP *vs.* CT**		▼	▲	▼	▲	▼	▲	▼
**PCPNIC *vs.* CT**		▼	▲	▼	▲	▼	▲	▼
Nicotinate metabolism (R-MMU-196807)	1						■	
Formation of Fibrin Clot (Clotting Cascade) (R-MMU-140877)	1		■					
Signaling by SCF-KIT (R-MMU-1433557)	1							■
Interleukin-2 family signaling (R-MMU-451927)	1							■
Signaling by Non-Receptor Tyrosine Kinases (R-MMU-9006927)	1							■
Signaling by PTK6 (R-MMU-8848021)	1							■
Signaling by PDGF (R-MMU-186797)	1							■
Arachidonic acid metabolism (R-MMU-2142753)	1			■				
PKR-mediated signaling (R-MMU-9833482)	1							■
Signaling by MET (R-MMU-6806834)	1							■
RHOC GTPase cycle (R-MMU-9013106)	1		■					
Mitochondrial protein degradation (R-MMU-9837999)	1					■		
Antiviral mechanism by IFN-stimulated genes (R-MMU-1169410)	1							■
Protein localization (R-MMU-9609507)	1					■		

UNCLASSIFIED (3 genes): Actr3b (▲,▲,▲), Sncb (▼,▼,▼), Calb2 (▲,▲,▲)
